# Supplementary material for: Trends in healthcare use in children aged less than 15 years: a population-based cohort study in England from 2007 to 2017
Source: BMJ Open. 2020 May 5;10(5):e033761. doi: 10.1136/bmjopen-2019-033761 (PMC7228511; doi:10.1136/bmjopen-2019-033761)
Supplement: Supplementary data [file bmjopen-2019-033761supp001.pdf]

Supplementary File 1

Creating GP consultations
We defined a GP consultation as any face to face consultation for illness that took place on practice premises, excluding consultations for routine preventive care (immunisation and development checks).

Using CPRD clinical data we:
1) Defined a GP consultation as direct contact between a GP and a child using the consultation type look up table COT if the event date was recorded. Direct consultations were for Clinic, Follow-up/routine visit, Surgery consultation, Acute visit, Emergency Consultation and Co-op Surgery Consultation. Thus, selected Constype = (1, 3, 9, 11, 18, 36).

2) Differentiated between consultation with a GP and consultation with another practice member of staff by highlighting the staff who were GPs using the staff id and the Role of staff look up table ROL. Roles classified as GP were Senior Partner, Partner, Assistant, Associate, Locum, GP Registrar, Salaried Partner and GP Retainer. Thus, selected Role code = (1, 2, 3, 4, 7, 8, 47, 50).

3) Excluded records that were administrative using the Consultation type look up table SED. Thus, excluded type = administration (6).

4) We excluded GP consultations for preventive care (using the patient id and consultation ids isolated in preventive care records below). Thus, excluded consultations for immunisations, child development check and routine child health check.

Supplementary Table 1: Read Codes identifying preventive care consultations

Description	V2 Read Codes
**Preventive care**	
Child development checks	64…
Routine child health check	ZV202
Vaccinations	All vaccinations (from CPRD immunization files)

Creating Emergency Department (ED) visits
An ED visit was defined as an emergency department attendance at a consultant-led emergency department with 24-hour service, full resuscitation facilities and designated accommodation for the reception of accident & emergency (A&E) patients and into other types of A&E/minor injury department. We excluded visits to consultant-led mono specialty A&E service such as specialist emergency eye units and NHS walk-in centres.

Using HES Accidents & Emergency data we:
1) Defined an ED visit using the field ‘AEDEPTTYPE’ if ‘ARRIVALDATE’ was recorded. Thus, selected ‘AEDEPTTYPE’ = (1,3)

2) Defined source of referral using the field ‘AEREFSOURCE’. Thus, selected self-referrals = ‘AEREFSOURCE’ = (1)

Creating Emergency and Elective admissions

Emergency admissions are unpredictable and occur at short notice because of clinical need whereas elective admissions are defined as occurring when the decision to admit can be separated in time from the actual admission.

Using HES Admitted Patient Care data we:

1) Defined an admission using the method of admission field 'ADMIMETH' if 'ADMIDATE' was recorded.

2) Differentiated between emergency admissions and elective admissions using the method of admission field 'ADMIMETH' below.

Supplementary Table 2: ADMIMETH codes identifying emergency and elecive admissions

Outcome	ADMIMETH code used
Emergency admissions	21, 22, 23, 24, 25, 28, 2A, 2B, 2C, 2D
Elective admissions	11, 12, 13

Creating outpatient visits

We defined an outpatient attendance where a child was recorded as having been seen by the intended care professional on the date of appointment on the HES outpatient appointments dataset.

Using HES Outpatients data we:

1) Defined an attendance to Outpatients using the attended or did not attend field 'ATTENDED' if the field 'APPTDATE' for the appointment date was recorded. Thus, selected 'ATTENDED' = (5, 6).
