# Supplementary material for: Trends in healthcare use in children aged less than 15 years: a population-based cohort study in England from 2007 to 2017
Source: BMJ Open. 2020 May 5;10(5):e033761. doi: 10.1136/bmjopen-2019-033761 (PMC7228511; doi:10.1136/bmjopen-2019-033761)
Supplement: Author's manuscript [file bmjopen-2019-033761.draft_revisions.pdf]

BMJ Open is committed to open peer review. As part of this commitment we make the peer review history of every article we publish publicly available.

When an article is published we post the peer reviewers' comments and the authors' responses online. We also post the versions of the paper that were used during peer review. These are the versions that the peer review comments apply to.

The versions of the paper that follow are the versions that were submitted during the peer review process. They are not the versions of record or the final published versions. They should not be cited or distributed as the published version of this manuscript.

BMJ Open is an open access journal and the full, final, typeset and author-corrected version of record of the manuscript is available on our site with no access controls, subscription charges or pay-per-view fees (http://bmjopen.bmj.com).

If you have any questions on BMJ Open's open peer review process please email info.bmjopen@bmj.com

BMJ Open

# Trends in healthcare use in children aged less than 15 years; a population-based cohort study in England from 2007 to 2017



| | |
|---|---|
| Journal: | *BMJ Open* |
| Manuscript ID | bmjopen-2019-033761 |
| Article Type: | Research |
| Date Submitted by the Author: | 21-Aug-2019 |





| | |
|---|---|
| Complete List of Authors: | Ruzangi, Judith; Imperial College London,<br>Blair, Mitch; Imperial College London, Paediatrics<br>Cecil, E; Imperial College London, Primary Care and Public Health<br>Greenfield, Geva; Imperial College London, Department of Primary Care & Public Health<br>Bottle, Alex; Imperial College, Primary Care and Social Medicine<br>Hargreaves, Dougal; Imperial College London, Primary Care and Public Health<br>Saxena, Sonia; Imperial College London, School of Public Health; |





| | |
|---|---|
| Keywords: | Healthcare use, GP consultations, Emergency department visits, Emergency admissions, Children |
| | |



SCHOLARONE™
Manuscripts

*I, the Submitting Author has the right to grant and does grant on behalf of all authors of the Work (as defined in the below author licence), an exclusive licence and/or a non-exclusive licence for contributions from authors who are: i) UK Crown employees; ii) where BMJ has agreed a CC-BY licence shall apply, and/or iii) in accordance with the terms applicable for US Federal Government officers or employees acting as part of their official duties; on a worldwide, perpetual, irrevocable, royalty-free basis to BMJ Publishing Group Ltd ("BMJ") its licensees and where the relevant Journal is co-owned by BMJ to the co-owners of the Journal, to publish the Work in this journal and any other BMJ products and to exploit all rights, as set out in our licence.*

*The Submitting Author accepts and understands that any supply made under these terms is made by BMJ to the Submitting Author unless you are acting as an employee on behalf of your employer or a postgraduate student of an affiliated institution which is paying any applicable article publishing charge ("APC") for Open Access articles. Where the Submitting Author wishes to make the Work available on an Open Access basis (and intends to pay the relevant APC), the terms of reuse of such Open Access shall be governed by a Creative Commons licence – details of these licences and which Creative Commons licence will apply to this Work are set out in our licence referred to above.*

*Other than as permitted in any relevant BMJ Author's Self Archiving Policies, I confirm this Work has not been accepted for publication elsewhere, is not being considered for publication elsewhere and does not duplicate material already published. I confirm all authors consent to publication of this Work and authorise the granting of this licence.*

For peer review only

# Trends in healthcare use in children aged less than 15 years; a population-based cohort study in England from 2007 to 2017

Judith Ruzangi,[1]* Mitch Blair,[2] Elizabeth Cecil, [1] Geva Greenfield, [1] Alex Bottle, [1] Dougal Hargreaves, [1] Sonia Saxena, [1]

Department of Primary Care & Public Health, School of Public Health, Imperial College London

***Corresponding author**: Judith Ruzangi;
Department of Primary Care and Public Health, School of Public Health, Imperial College London, 319 The Reynolds Building, St Dunstan's Road, London W6 8RP j.ruzangi@imperial.ac.uk

## Institutional affiliations

1. Department of Primary Care and Public Health, School of Public Health, Imperial College London. London. UK
2. River Island Academic Centre for Paediatrics and Child Health Imperial College London. London. UK Northwick Park Hospital Campus

## Guarantor

Professor Sonia Saxena: ORCID 0000-0003-3787-2083

Manuscript word count: 3097

For peer review only

# Abstract

## Objective

To describe changing use of primary care in relation to use of urgent care and planned hospital services by children aged less than 15 years in England in the decade following major primary care reforms from 2007-17

## Design

Population based retrospective cohort study.

## Methods

We used linked data from the Clinical Practice Research Datalink to study children's primary care consultations, emergency department visits, emergency and elective admissions to hospital and outpatient visits.

## Results

Between 1 April 2007 to 31 March 2017 there were 7,604,024 GP consultations, 981,684 ED visits, 287,719 emergency hospital admissions, 2,253,533 outpatient visits and 194,034 elective admissions among 1,484,455 children aged less than 15 years. Age standardised GP consultation rates fell (-1.0% /year) to 1864 per 1000 child-years in 2017 in all age bands except infants rising by 1%/ year to 6722 per 1000/ child-years in 2017. ED visit rates increased by 1.6%/ year to 369 per 1000 child-years in 2017, with steeper rises of 3.9%/year in infants (780 per 1000 child-years in 2017). Emergency hospital admission rates rose steadily by 3%/ year to 86 per 1000 child-years and outpatient visit rates rose to 724 per 1000 child-years in 2017.

## Conclusions

Over the past decade since NHS primary care reforms, GP consultations have fallen for all children, except for infants. Children's use of hospital urgent and outpatient care has risen in all ages, especially infants. These changes signify the need for better access and provision of specialist and community-based support for families with young children.


Keywords: Healthcare use, Children, GP consultations, Emergency department visits, Emergency admissions

For peer review only

## Strengths and Limitations

- This is the largest nationally representative population-based study to date examining trends in children's healthcare use in the National Health Service in England
- The cohort design using individual level linked data from primary care and hospitals enabled a whole systems analysis of children's health care use
- Previous studies focused on children's primary care use have not previously been reported
- Improvements in data quality have enabled analysis of children's emergency department visit and outpatient visits
- The study population was children registered with a general practice, which underrepresents those from poorer postcodes, mobile or homeless populations
- We restricted our analysis to data from England due to limitations of linking data from primary care and hospitals in other regions in the United Kingdom

For peer review only

## Background

Primary care is at the heart of effective health systems but globally both primary care and hospital services are increasingly under pressure (1). The strengths of primary care include comprehensive, coordinated, preventive and planned care that has been shown to improve population health and reduce avoidable health system waste (2). In the United Kingdom (UK) National Health Service (NHS), over 98% of children are registered from birth with a UK general practitioner (GP) and receive preventive care including vaccinations and development checks as well as first contact care for acute problems (3). Infants and preschool children have among the highest visit rates of any age group to primary care (1). GPs also diagnose and manage chronic illnesses that emerge across the life course and play a crucial role in prioritising referral on for specialist care in hospital and outpatient settings. This cradle to grave model has been in place since the creation of the NHS over 70 years ago. However, major reforms in NHS primary care over the past decade are thought to have pushed clinical and administrative GP workloads to saturation point and reduced children's access to GPs in and out of hours. From 2004, most GPs opted out of providing out of hour's services and took on a greater role in active monitoring and management of a rising population burden of chronic conditions in adults, driven by financial incentive schemes. Face to face GP consulting rates increased by 10% and telephone consulting rates doubled in the decade to 2013/14.

There is some evidence that children's use of hospital emergency services is sensitive to access and availability of primary care. In the period following primary care reforms of 2004, there were large increases in children's visits to emergency departments (ED) and rises in short stay hospital admissions for primary care sensitive conditions (4). A third of all British children aged less than 15 years currently visit ED each year (5). This figure rises to 40% in practices where access is poor (3). Two-thirds of children visit outside of normal GP consulting hours although the peak time for ED visits is between 4 and 6pm (3). A relatively high proportion of children visiting ED (15-80%) are discharged with no treatment or present with conditions that could potentially have been treated in primary care (6,7). Previous ecological studies have been unable to provide population-based estimates of children's health service use using individual level data across the primary and secondary care interface. The aim of this study was to describe trends in use of primary care in relation to use of urgent care and planned NHS hospital services by children aged less than 15 years in England in the decade following major primary care reforms.

## Methods

### Study design and data sources

We carried out a cohort study using prospectively collected data from the Clinical Practice Research Datalink (CPRD), which is the largest validated primary care research database within the UK (8). It contains longitudinal, patient-level, anonymised computerised health records from more than 600 participating UK general practices (GPs) for 22 million patients (8). It is representative of the general UK population with respect to age, sex and ethnicity and covers approximately 8% of that population (8,9). We used CPRD linked to Hospital Episodes Statistics (HES) to obtain information on ED visits, emergency hospital admissions, elective admissions and outpatient attendance. HES contain information on NHS hospital activity in England. Ethical approval for this study was obtained from the Independent Scientific Advisory Committee (ISAC), which oversees research involving CPRD data (Protocol 18_139).

### Population

We constructed an open cohort including all children aged less than 15 years in HES linked CPRD registered "up to standard" CPRD-participating general practices during the study period from 1/04/2007 to 31/07/2017. Each child contributed to time of observation from birth or first registration date. Follow up for each year was continuous for each child until either the date they transferred out



For peer review only

of practice, reached the age of 15 years, died or reached the end of the study period (31/07/2017), whichever came first.

Children were assigned to one of 4 developmental age groups; infants aged < 1-year, preschool children aged 1–4 years, school children aged 5–9 years and teenagers aged 10-14 years. Since CPRD data does not contain birth dates, we ascribed a birth date of the 1st of the recorded month and year of birth to each child. For children whose record had missing data for the month of their birth, we assumed a birthdate of the 1st Jan on their year of birth. Children contributed data on health service use at different ages across each year of the study period. For each financial year, age for each child was defined as the number of years since the birthdate of that child to the end of that financial year (31 March). Hence, this was an open cohort with children entering and leaving the sample as well as changes in age across the decade.

## Outcomes

Our main outcomes were age standardised rates for GP consultation, ED visits, emergency and elective hospital admissions and outpatient visits (10). We defined a GP consultation as any face to face consultation for illness that took place on practice premises, excluding consultations for routine preventive care (immunisation and development checks). An ED visit was defined as an emergency department attendance at a consultant-led emergency department with 24-hour service, full resuscitation facilities and designated accommodation for the reception of accident & emergency (A&E) patients and into other types of A&E/minor injury department. We excluded visits to consultant-led mono specialty A&E service such as specialist emergency eye units and NHS walk-in centres. Emergency admissions were admissions that were ‘unpredictable and occur at short notice because of clinical need’, whereas elective admissions were defined as occurring when the ‘decision to admit can be separated in time from the actual admission’. We defined an outpatient attendance where a child was recorded as having been seen by the intended care professional on the date of appointment on the HES outpatient appointments dataset (10). These outcomes are described in Supplementary File 1.

## Analysis

We calculated annual rates per 1000 child-years for each outcome for each financial year by summing the total number of events divided by the total child years of observation for all children aged less than 15 years. To enable comparison across years, rates were age standardised to the 2016 mid-year English population aged<15 years (11). Similarly, annual rates were calculated for each developmental age-group from 2007 to 2017. However, due to poor HES A&E data quality before 2011, we only report ED visit rates from 2011/2012 (12). We calculated as the change from baseline rate in 2007/8 to 2016/17, except for ED visits where we used a baseline reference year of 2011/12. We calculated the proportion of children with an ED visit who self-referred. We calculated the conversion rate for emergency admission as the proportion of ED visits that led to emergency admissions.

# Results

Overall, 1,484,455 children from 408 practices contributed to this 10-year cohort study.

From 1 April 2007 to 31 March 2017, there were 7,604,024 GP consultations, 981,684 ED visits, 287,719 emergency admissions, 194,034 elective admissions, 2,253,533 outpatient visits among children aged less than 15 years in our population (Figure 1).

## GP consultations

The age standardised GP consultation rate in children less than 15 years dropped from 2005 to 1864 per 1000 child-years between 2007/8 and 2016/17 (Figure 2). The GP consultation rate in infants increased by 1%/ year from 6084 per 1000 child-years in 2007/2008 to 6722 per 1000 child-years in



For peer review only

2016/2017. The GP consultation rate in pre-school children and those aged 5-9 years fell from 2707 to 2368 per 1000 child-years and 1416 to 1217 per 1000 child-years in the same study period respectively. For children aged above 10 years, the rate was stable throughout the decade (1100 per 1000 child-years) (Table 1).

For peer review only

**Table 1 Annual rates of healthcare use per 1000 child-years and relative change from baseline 2007-2017[1,2]**

| | Infants < 1 years | | | Preschool children 1-4 years old | | | School children 5-9 years old | | | Teenagers 10-14 years old | | |
|---|---|---|---|---|---|---|---|---|---|---|---|---|
| | **2007/08** | **2016/17** | **% change[1] (95% CI)** | **2007/08** | **2016/17** | **% change[1] (95% CI)** | **2007/08** | **2016/17** | **% change[1] (95% CI)** | **2007/08** | **2016/17** | **% change[1] (95% CI)** |
| **General practice consultations** | 6084 | 6722 | 10.5 (8.9, 12.0) | 2707 | 2368 | -12.5 (-13.2, -11.8) | 1416 | 1218 | -14.0 (-14.8, -13.2) | 1143 | 1074 | -6.0 (-7.0, -5.0) |
| **Emergency admissions** | 312 | 410 | 31.6 (24.2, 39.1) | 109 | 123 | 12.8 (8.8, 16.8) | 38 | 39 | 2.8 (-2.9, 8.5) | 34 | 34 | -1.4 (-8.1, 5.2) |
| **Elective admissions** | 47 | 68 | 46.4 (14.1, 78.6) | 53 | 52 | -3.0 (-13.7, 7.6) | 48 | 47 | -2.4 (-10.3, 5.5) | 38 | 46 | 20.7 (-1.0, 42.3) |
| **Outpatient visits** | 883 | 1529 | 73.1 (66.0, 80.2) | 518 | 706 | 36.3 (33.7, 38.9) | 477 | 649 | 36.2 (33.8, 38.6) | 451 | 648 | 43.9 (41.1, 46.7) |
| | **2011/12** | **2016/17** | **% change (95% CI)** | **2011/12** | **2016/2017** | **% change (95% CI)** | **2011/12** | **2016/17** | **% change (95% CI)** | **2011/12** | **2016/17** | **% change (95% CI)** |
| **Emergency department visits[2]** | 562 | 780 | 38.6 (32.8, 44.5) | 419 | 497 | 18.8 (17.0, 20.5) | 226 | 249 | 10.3 (8.2,12.3) | 280 | 297 | 6.2 (4.2, 8.1) |

1. % change calculated as (rate in 2016/2017 – rate in 2007/2008)/rate in 2007/2008; 95% confidence interval (CI)
2. % change in emergency department visits calculated from baseline year starts from 2011/2012 due to poor data quality poor prior to this time

For peer review

### Urgent and emergency care; ED visits and emergency hospital admissions

From 2011 to 2017, the age standardised ED visit rate in children aged less than 15 years increased by 1.6%/ year from 319 to 369 per 1000 child-years. There was a much larger increase of 3.9%/ year in annual ED visit rate for infants compared with older children (562 to 780 per 1000 child-years from 2011 to 2017). Since 2011, every year, 70% of children visiting ED self-referred and 22% of ED visits led to an emergency admission. From 2007/2008 to 2016/2017, the age standardised emergency admission rate in children less than 15 years increased by 1.5%/ year from 75 to 86 per 1000 child-years and for infants by 3%/ year from 312 to 410 per 1000 child-years. However, the emergency admission rate in pre-school children and those aged above 5 years was stable throughout the decade (100-130 and <50 per 1000 child-years respectively).

### Planned care; outpatient visits and elective admissions

From 2007/8 to 2016/17, the annual age standardised outpatient visit rate in children aged less than 15 years increased from 508 to 724 per 1000 child-years. The outpatient visit rate in infants increased by 7.3%/ year from 883 to 1529 per 1000 child-years over the study period. Rates in pre-school children and those aged above 5 years also increased steadily throughout the same period from 500 to 700 per 1000 child-years (Figure 3). Age standardised elective admission rates in children less than 15 years over the same study period were stable from 46 to 49 per 1000 child-years but increased by 4.6%/ year from 47 to 68 per 1000 child-years for infants and by 2%/ year for teenagers from 38 to 46 per 1000 child-years. Rates were stable for pre-schoolers and school children (~50 per 1000 child-years).

## Discussion

### Key findings

Infant contact rates with GPs have increased year on year over the past decade following major reforms in primary care but older children are using primary care less. Children's use of urgent care in hospitals, including visits to EDs and emergency admission rates, have increased particularly among infants. There have been sharp increases in planned care including outpatient activity and admissions for elective care in infants and teenage children.

### Comparison with previous studies

We have previously reported GP consultation rates for illness in children of a similar magnitude for a CPRD cohort born in 2000 and another study of healthcare use in primary care study  that are remarkably consistent with our findings here for children (1,13). Infants, however, seem to be seeing their GP more frequently in 2017 compared with a decade ago. The expansion in the use of urgent care is consistent with trends in the UK and other high-income country settings where emergency admission rates in both children and adults have been rising for several years (4,14). There is little research on children's outpatient service use and elective admissions but are consistent with overall rises in the total number of elective admissions, referrals to outpatient services and total outpatient attendances in English hospitals, which has increased markedly since 2007 (15–17).

Rising needs in the child population and demand from families are most likely contributors to the patterns of overall increasing healthcare use by children seen here. Preterm birth is increasing and is the single biggest cause of neonatal morbidity and mortality in the UK and other countries where survival rates are increasing (18). Thus, rising emergency admissions in infants could be explained by a growing morbidity burden in infants compared with other children. Recurrent admissions in children and young people with chronic conditions contribute substantially to total emergency admissions, affecting (19). A report projecting the State of Child Health in 2030 concluded that Britain's children who make up nearly 20% of the population have a rising health burden and poorer health compared

For peer review only

with other wealthy countries. Even more concerning is a widening in the gap in health between wealthy and poor children (20). Hence growing morbidity burden particularly in deprived groups may be contributing to the rise in health care use.

Health system failures to provide responsive care in the community to meet the needs of the children and families might well explain the rise in urgent care use. Parents prefer using their regular general GP when their child is unwell, but they choose to visit the ED if they perceive their child's condition as serious or cannot access primary care (21–23). We found the majority (70%) of visits were self-referred and 80% of children discharged after their ED visit. It is estimated that 10% of infants attending the ED have no underlying medical problem (24), yet this does not mean that 90% need to seek emergency care if they require support to care for their child. The 2004 primary care healthcare reforms focused heavily on improving chronic disease management in adults with long term conditions and allowed primary care physicians to opt out of providing acute primary care services during evenings and weekends. This reduced access that may explain rising emergency department visits (25). The introduction of four-hour waiting target in ED in 2004 has been previously suggested as a contributing factor to increases in emergency admission rates (26,27). However, less than 5% of children's admission breach the 4-hour waiting time target so pressures within EDs are unlikely to explain the rising admission rate (28). Similarly, there is little evidence that ED staff have lowered their threshold for admitting children in our findings since the attendance admission ratio remained around 1 in 5 across the decade. Thus, our findings and previous studies point to mounting pressures in the community and illustrate the remarkable ability of hospitals in England to respond to the needs of families with young children.

## Strengths and Limitations

Our study is the first to report on trends in healthcare use by children for both primary and hospital care in the UK. This is among the largest studies to report healthcare use in children across the primary and secondary care interface in a health system with universal health coverage. The strengths of this study include its size, national coverage and representativeness of the UK population of both CPRD and HES data sources widely used for research. This reduces the possibility that our findings are due to chance. Few countries have health systems that enable the study of individual level data on children and links between primary care and hospital data.

However, there are several limitations to consider. First, in common with many observational studies, data quality is variable. Use of HES A&E data was initiated in 2007/2008 but data quality and coverage did not improve until 2009. Hence we restricted our analysis of EDs to the period from 2011 onwards (12). HES Outpatient data were first available in 2003/2004 and has been validated. The rate of outpatient attendances in 2015/2016 has been ascribed to fluctuation in data quality and thus has not been reported in this paper (17). Second, we used a proxy for birthdate as CPRD only provides month of birth for children aged less than 15 years. Third, children living in deprived areas are more likely to deregister from CPRD practices than children residing in more affluent postcodes. Hence, our findings may not be generalisable to these groups (29). Finally, our findings may not be generalisable to certain patient groups missing from CPRD such as children using private health care services and homeless children (8).

## Implications and future research

The large sustained rises we report in primary care, urgent and planned care in hospital in infants have important implications for primary care. If rising urgent care use and falling contact rates with general practitioners are evidence of difficulties in children's access to GP appointments this is an important area for reform. It brings into question the continued selective financial incentive schemes for general practitioners to manage a rising multimorbidity burden in older patients. It is of concern that general practitioners are not universally trained in paediatrics and child health medicine as has

For peer review only

been the recommendation of the European Academy of Paediatrics recently (30). Regardless, upwards trends in planned and urgent healthcare use by children have major resource and cost implications for the NHS and we recommend the drivers and mediators of these changes warrant further research (25,31,32). A further breakdown of data, which includes ethnic background, index of multiple deprivation, repeat attendance, presenting problems and case mix would be immensely helpful in understanding these trends. The number of health visitors and midwives who are providing support has decreased in England and Wales in recent years as a result of funding cuts (33). Accurate data on the effectiveness of this workforce in supporting parents is currently lacking but is recommended as an area for future research. Finally, there is scope to explore innovative approaches including parenting programmes and peer support for increasing parent's confidence and capacity in caring for their children.

## Conclusions

Over the past decade in England, general practice consultations have fallen for all children, except for infants. Children's use of hospital urgent and outpatient care has risen in all ages, especially infants. These changes may signify the need for better access to primary care and provision of specialist and community-based support for families with young children. Research to understand drivers and identify solutions to rising health service use is urgently needed.

For peer review only

## References

1. Hobbs FDR, Bankhead C, Mukhtar T, Stevens S, Perera-Salazar R, Holt T, et al. Clinical workload in UK primary care: a retrospective analysis of 100 million consultations in England, 2007–14. Lancet. 2016;387(10035):2323–30.
2. Shi Leiyu MJSB. Contribution of primary care to health systems and health. Milbank Q. 2005 Sep 1;83(3):457–502.
3. Cecil E, Bottle A, Cowling TE, Majeed A, Wolfe I, Saxena S. Primary Care Access, Emergency Department Visits, and Unplanned Short Hospitalizations in the UK. Pediatrics. 2016 Feb 1;137(2):e20151492.
4. Cecil E, Bottle A, Sharland M, Saxena S. Impact of UK Primary Care Policy Reforms on Short-Stay Unplanned Hospital Admissions for Children With Primary Care–Sensitive Conditions. Ann Fam Med. 2015;13(3):214.
5. Keeble E, Kossarova L. Focus on: Emergency hospital care for children and young people. 2017.
6. Smith L, Narang Y, Ibarz Pavon AB, Edwardson K, Bowers S, Jones K, et al. To GP or not to GP: a natural experiment in children triaged to see a GP in a tertiary paediatric emergency department (ED). BMJ Qual Saf. 2017 Sep;bmjqs-2017-006605.
7. Gnani S, Morton S, Ramzan F, Davison M, Ladbrooke T, Majeed A, et al. Healthcare use among preschool children attending GP-led urgent care centres: a descriptive, observational study. BMJ Open. 2016 Jun;6(6):e010672.
8. Herrett E, Gallagher AM, Bhaskaran K, Forbes H, Mathur R, Van Staa T, et al. Data Resource Profile: Clinical Practice Research Datalink (CPRD).
9. Mathur R, Bhaskaran K, Chaturvedi N, Leon DA, Van Staa T, Grundy E, et al. Completeness and usability of ethnicity data in UK-based primary care and hospital databases. J Public Heal (United Kingdom). 2014;36(4):684–92.
10. Hospital Episode Statistics Data Dictionary - NHS Digital [Internet]. [cited 2019 May 31].
11. Population estimates for the UK, England and Wales, Scotland and Northern Ireland - Office for National Statistics [Internet]. [cited 2019 Jun 7].
12. HES 2011-12 A&E Data Quality Note Experimental data set. 2013.
13. Cecil E, Bottle A, Ma R, Hargreaves DS, Wolfe I, Mainous AG, et al. Impact of preventive primary care on children's unplanned hospital admissions: a population-based birth cohort study of UK children 2000–2013. BMC Med. 2018 Dec 17;16(1):151.
14. Health at a Glance 2015: OECD Indicators. Paris: OECD Publishing; 2015.
15. Baker C. NHS Key Statistics: England, May 2019.
16. Dreyer K, Williamson RAP, Hargreaves DS, Rosen R, Deeny SR. Associations between parental mental health and other family factors and healthcare utilisation among children and young people: a retrospective, cross-sectional study of linked healthcare data. BMJ Paediatr Open. 2018 Jul 30;2(1):e000266.
17. Hospital Outpatient Activity - NHS Digital [Internet]. [cited 2019 May 31].
18. Blencowe H, Cousens S, Oestergaard MZ, Chou D, Moller A-B, Narwal R, et al. National, regional, and worldwide estimates of preterm birth rates in the year 2010 with time trends since 1990 for selected countries: a systematic analysis and implications. Lancet (London, England). 2012 Jun 9;379(9832):2162–72.
19. Wijlaars LP, Hardelid P, Woodman J, Allister J, Cheung R, Gilbert R. Contribution of recurrent admissions in children and young people to emergency hospital admissions: retrospective cohort analysis of hospital episode statistics. Arch Dis Child. 2015 Sep;100(9):845–9.
20. Viner R, Ward J, Cheung R, Wolfe I, Hargreaves D. Child health in 2030 in England: comparisons with other wealthy countries.
21. Williams A, O'Rourke P, Keogh S. Making choices: why parents present to the emergency department for non-urgent care. Arch Dis Child. 2009 Oct 1;94(10):817–20.
22. Woolfenden S, Ritchie J, Hanson R, Nossar V. Parental use of a paediatric emergency department as an ambulatory care service. Aust N Z J Public Health. 2000 Apr 1;24(2):204–6.
23. Maguire S, Ranmal R, Komulainen S, Pearse S, Maconochie I, Lakhanpaul M, et al. Which urgent care services do febrile children use and why? Arch Dis Child. 2011 Sep 1;96(9):810–6.
24. Heys M, Kwong H-M, Reed J, Blair M. What do we really know about infants who attend Accident and Emergency departments? Perspect Public Health. 2014 Mar;134(2):93–100.
25. Cecil E, Bottle A, Cowling TE, Majeed A, Wolfe I, Saxena S. Primary Care Access, Emergency Department Visits, and Unplanned Short Hospitalizations in the UK. Pediatrics. 2016 Feb;137(2):e20151492.

For peer review only

26. Saxena S, Bottle A, Gilbert R, Sharland M. Increasing Short-Stay Unplanned Hospital Admissions among Children in England; Time Trends Analysis '97–'06. van Baal PHM, editor. PLoS One. 2009 Oct;4(10):e7484.
27. Gill PJ, Goldacre MJ, Mant D, Heneghan C, Thomson A, Seagroatt V, et al. Increase in emergency admissions to hospital for children aged under 15 in England, 1999–2010: national database analysis. Arch Dis Child. 2013 May 1;98(5):328–34.
28. Blunt I. Focus on : A & E attendances.Why are patients waiting longer. 2014.
29. Howe LD, Tilling K, Galobardes B, Lawlor DA. Loss to follow-up in cohort studies: bias in estimates of socioeconomic inequalities. Epidemiology. 2013 Jan;24(1):1–9.
30. Završnik J, Stiris T, Schrier L, Russell RR, del Torso S, Valiulis A, et al. Basic training requirements for health care professionals who care for children. Eur J Pediatr. 2018 Sep 25;177(9):1413–7.
31. Watson M, Blair M. Emergency departments and minor illness: Some behavioural insights. Archives of Disease in Childhood. 2018;103(4):309–10.
32. Shraim M, Blagojevic-Bucknall M, Mallen CD, Dunn KM. Repeated primary care consultations for non-specific physical symptoms in children in UK: a cohort study. BMC Fam Pract. 2014 Dec 5;15:195.
33. Buchan J, Charlesworth A, Gershlick B, Seccombe I. A critical moment: NHS staffing trends, retention and attrition. 2019.

For peer review only

# Additional Information

## Contributors:

SS and MB conceived and designed this study. JR prepared the data and carried out the statistical analysis overseen by EC and AB. All authors took part in interpreting the data for this study, commented on and helped to revise drafts of this paper and have approved the final version. SS is the guarantor

**Funding:** This article presents independent research supported by the National Institute for Health Research (NIHR) School for Public Health research (SS) the Collaborations for Leadership in Applied Health Research and Care (CLAHRC) programme for Northwest London (PD-SPH-2015-10055). The Dr Foster Unit at Imperial is affiliated with the National Institute of Health Research (NIHR) Imperial Patient Safety Translational Research Centre. The NIHR Imperial Patient Safety Translational Centre is a partnership between the Imperial College Healthcare NHS Trust and Imperial College London. The Dr Foster Unit at Imperial College are grateful for support from the NIHR Biomedical Research Centre funding scheme.

**Disclaimer:** The views expressed are those of the authors and not necessarily those of the NHS, the NIHR or the Department of Health and Social Care.

**Competing interests:** The authors declare that they have no competing interests.

**Patient consent:** Not required.

**Ethics approval:** We obtained ethical and scientific approval for the use of CPRD for our study from the Independent Scientific Advisory Committee Protocol number: 18_139.

**Provenance and peer review:** Not commissioned; externally peer reviewed.

**Data sharing Copyright information:** due to data sharing agreements, data are unavailable for sharing publicly.

**Copyright information:** This is an open access article distributed in accordance with the Creative Commons Attribution Non Commercial (CC BY-NC 4.0) license, which permits others to distribute, remix, adapt, build upon this work non-commercially, and license their derivative works on different terms, provided the original work is properly cited, appropriate credit is given, any changes made indicated, and the use is non-commercial. See: http://creativecommons.org/licenses/by-nc/4.0/.

For peer review only

Figure 1 Total healthcare use in children from 2007/2008 to 2016/2017

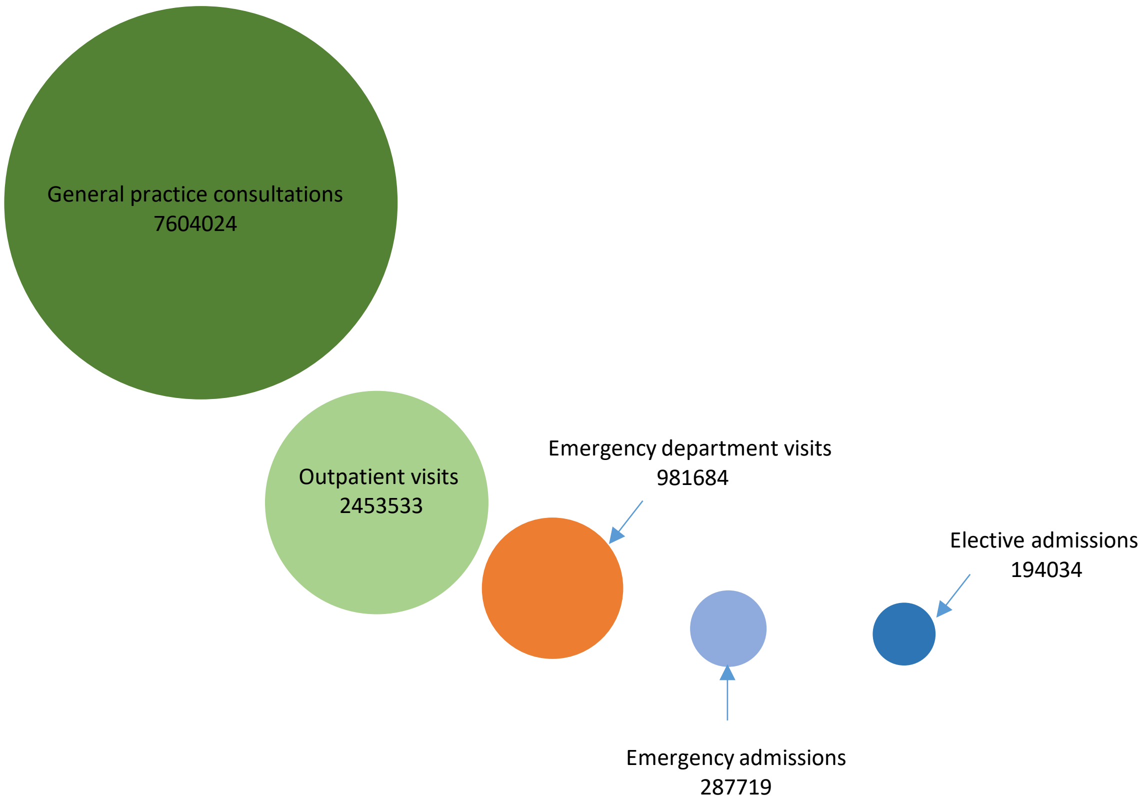


w only

Figure 2 Healthcare use in children aged less than 15 years

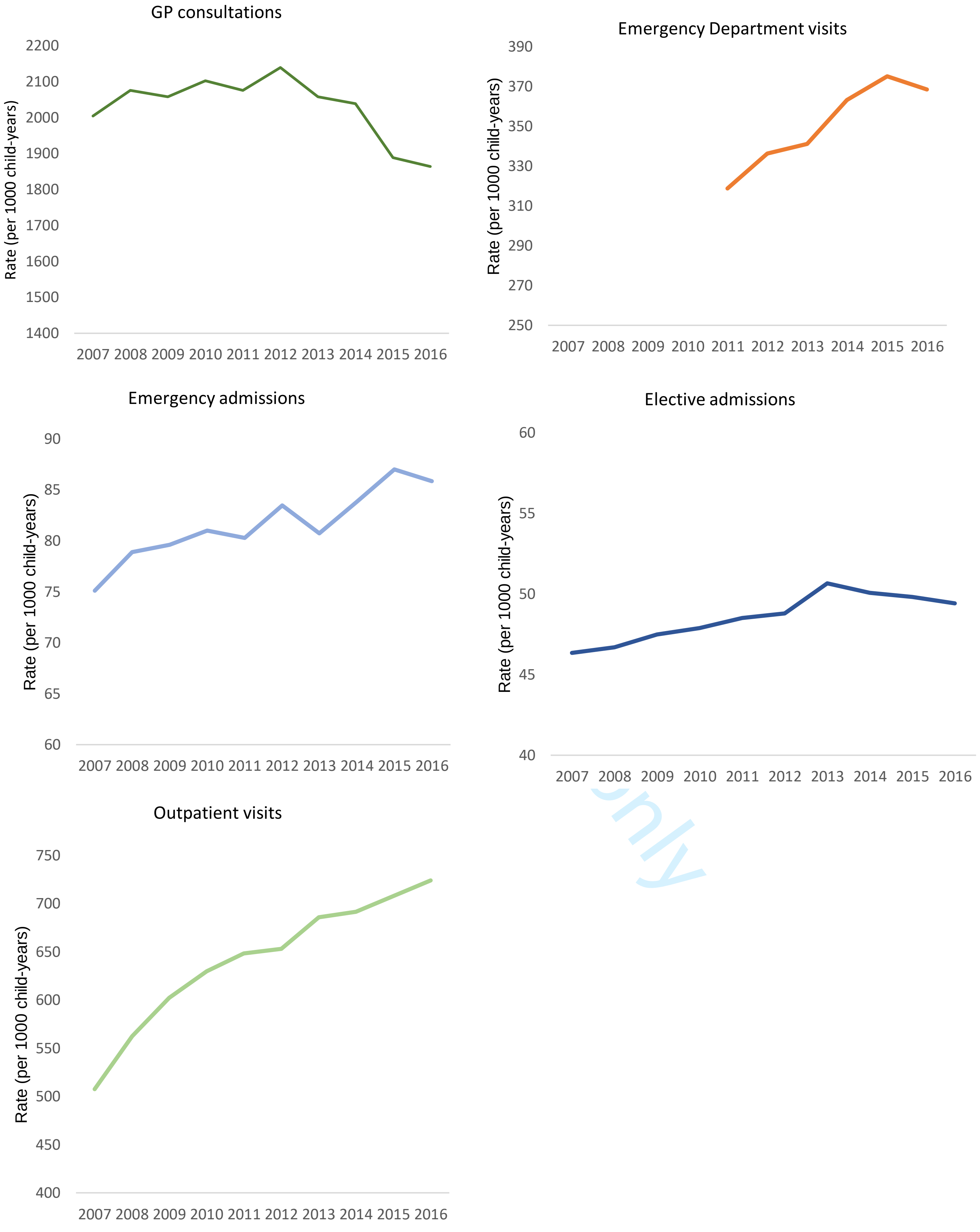


Rates were directly age-standardised to the 2016 mid-year English population

Figure 3 Healthcare use in children by developmental age groups

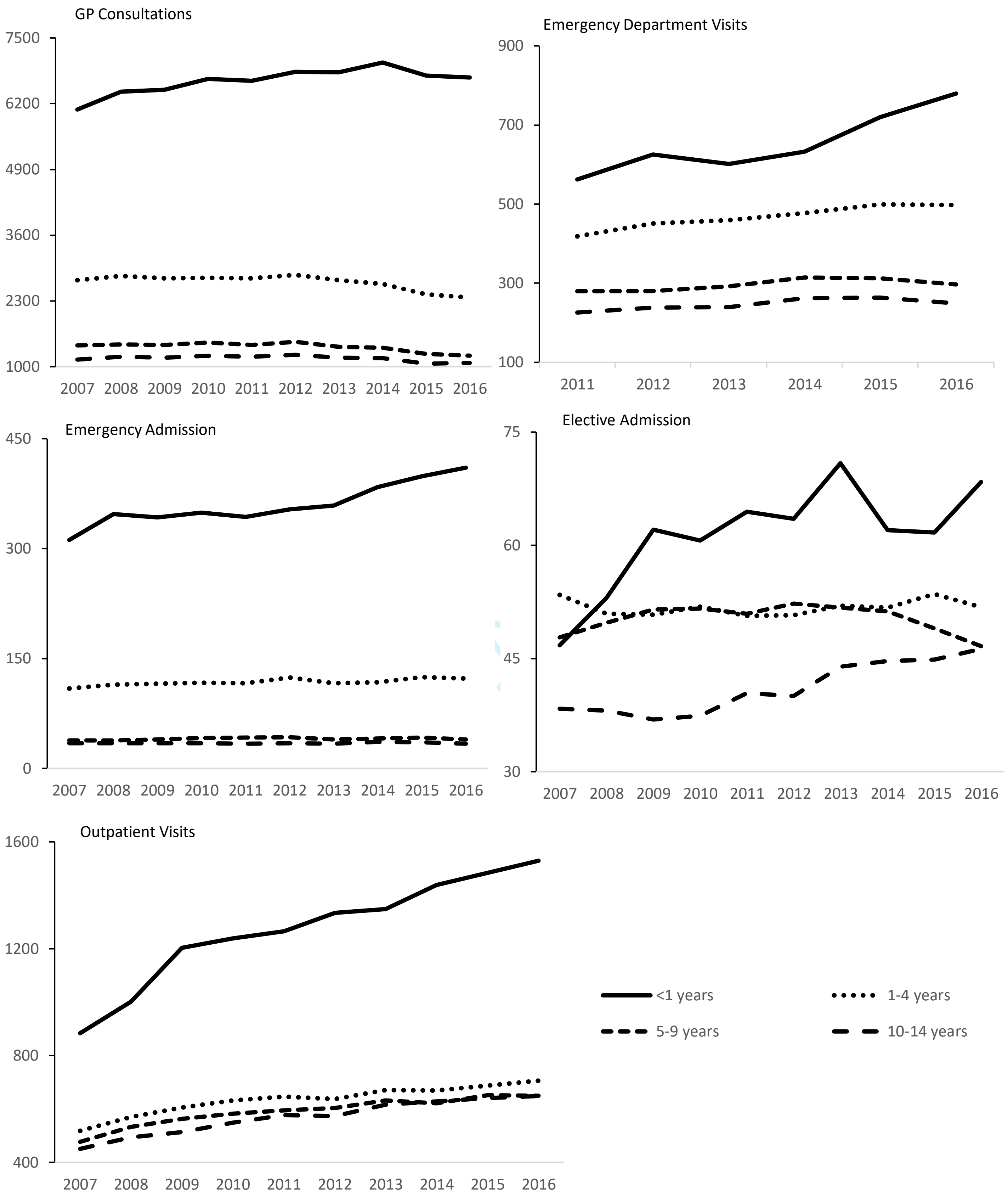

## Supplementary Methods

### Creating GP consultations

We defined a GP consultation as any face to face consultation for illness that took place on practice premises, excluding consultations for routine preventive care (immunisation and development checks).

Using CPRD clinical data we:

1) Defined a GP consultation as direct contact between a GP and a child using the consultation type look up table COT if the event date was recorded. Direct consultations were for Clinic, Follow-up/routine visit, Surgery consultation, Acute visit, Emergency Consultation and Co-op Surgery Consultation. Thus, selected Constype = (1, 3, 9, 11, 18, 36).

2) Differentiated between consultation with a GP and consultation with another practice member of staff by highlighting the staff who were GPs using the staff id and the Role of staff look up table ROL. Roles classified as GP were Senior Partner, Partner, Assistant, Associate, Locum, GP Registrar, Salaried Partner and GP Retainer. Thus, selected Role code = (1, 2, 3, 4, 7, 8, 47, 50).

3) Excluded records that were administrative using the Consultation type look up table SED. Thus, excluded type = administration (6).

4) We excluded GP consultations for preventive care (using the patient id and consultation ids isolated in preventive care records below). Thus, excluded consultations for immunisations, child development check and routine child health check.

### Supplementary Table 1: Read Codes identifying preventive care consultations

| Description | V2 Read Codes |
|---|---|
| **Preventive care** | |
| Child development checks | 64… |
| Routine child health check | ZV202 |
| Vaccinations | All vaccinations (from CPRD immunization files) |

### Creating Emergency Department (ED) visits

An ED visit was defined as an emergency department attendance at a consultant-led emergency department with 24-hour service, full resuscitation facilities and designated accommodation for the reception of accident & emergency (A&E) patients and into other types of A&E/minor injury department. We excluded visits to consultant-led mono specialty A&E service such as specialist emergency eye units and NHS walk-in centres.

Using HES Accidents & Emergency data we:

1) Defined an ED visit using the field ‘AEDEPTTYPE’ if ‘ARRIVALDATE’ was recorded. Thus, selected ‘AEDEPTTYPE’ = (1,3)

2) Defined source of referral using the field ‘AEREFSOURCE’. Thus, selected self-referrals = ‘AEREFSOURCE’ = (1)

For peer review only

**Creating Emergency and Elective admissions**
Emergency admissions are unpredictable and occur at short notice because of clinical need whereas elective admissions are defined as occurring when the decision to admit can be separated in time from the actual admission.

Using HES Admitted Patient Care data we:

1) Defined an admission using the method of admission field 'ADMIMETH' if 'ADMIDATE' was recorded.

2) Differentiated between emergency admissions and elective admissions using the method of admission field 'ADMIMETH' below.

**Supplementary Table 2: ADMIMETH codes identifying emergency and elecive admissions**

| Outcome | ADMIMETH code used |
|---|---|
| Emergency admissions | 21, 22, 23, 24, 25, 28, 2A, 2B, 2C, 2D |
| Elective admissions | 11, 12, 13 |

**Creating outpatient visits**
We defined an outpatient attendance where a child was recorded as having been seen by the intended care professional on the date of appointment on the HES outpatient appointments dataset.

Using HES Outpatients data we:

1) Defined an attendance to Outpatients using the attended or did not attend field 'ATTENDED' if the field 'APPTDATE' for the appointment date was recorded. Thus, selected 'ATTENDED' = (5, 6).

For peer review only

# Annex 1: STROBE Statement—checklist of items that should be included in reports of observational studies

| | Item No | Recommendation | Pg. |
|---|---|---|---|
| **Title and abstract** | 1 | (*a*) Indicate the study's design with a commonly used term in the title or the abstract | 1 |
| | | (*b*) Provide in the abstract an informative and balanced summary of what was done and what was found | 2 |
| **Introduction** | | | |
| Background/rationale | 2 | Explain the scientific background and rationale for the investigation being reported | 4 |
| Objectives | 3 | State specific objectives, including any prespecified hypotheses | 4 |
| **Methods** | | | |
| Study design | 4 | Present key elements of study design early in the paper | 4 |
| Setting | 5 | Describe the setting, locations, and relevant dates, including periods of recruitment, exposure, follow-up, and data collection | 4 |
| Participants | 6 | (*a*) *Cohort study*—Give the eligibility criteria, and the sources and methods of selection of participants. Describe methods of follow-up<br>*Case-control study*—Give the eligibility criteria, and the sources and methods of case ascertainment and control selection. Give the rationale for the choice of cases and controls | 4 |

For peer review only

| | | | |
|---|---|---|---|
| | | *Cross-sectional study*—Give the eligibility criteria, and the sources and methods of selection of participants | |
| | | (*b*) *Cohort study*—For matched studies, give matching criteria and number of exposed and unexposed<br>*Case-control study*—For matched studies, give matching criteria and the number of controls per case | |
| Variables | 7 | Clearly define all outcomes, exposures, predictors, potential confounders, and effect modifiers. Give diagnostic criteria, if applicable | 5 |
| Data sources/ measurement | 8* | For each variable of interest, give sources of data and details of methods of assessment (measurement). Describe comparability of assessment methods if there is more than one group | 5 |
| Bias | 9 | Describe any efforts to address potential sources of bias | 5 |
| Study size | 10 | Explain how the study size was arrived at | N/A |
| Quantitative variables | 11 | Explain how quantitative variables were handled in the analyses. If applicable, describe which groupings were chosen and why | 5 |
| Statistical methods | 12 | (*a*) Describe all statistical methods, including those used to control for confounding | 5 |
| | | (*b*) Describe any methods used to examine subgroups and interactions | N/A |
| | | (*c*) Explain how missing data were addressed | 5 |
| | | (*d*) *Cohort study*—If applicable, explain how loss to follow-up was addressed | N/A |

For peer review only

| | | | |
|---|---|---|---|
| | | *Case-control study*—If applicable, explain how matching of cases and controls was addressed<br>*Cross-sectional study*—If applicable, describe analytical methods taking account of sampling strategy | |
| | | (*e*) Describe any sensitivity analyses | N/A |
| **Results** | | | |
| Participants | 13* | (a) Report numbers of individuals at each stage of study—eg numbers potentially eligible, examined for eligibility, confirmed eligible, included in the study, completing follow-up, and analysed | 5 |
| | | (b) Give reasons for non-participation at each stage | N/A |
| | | (c) Consider use of a flow diagram | |
| Descriptive data | 14* | (a) Give characteristics of study participants (eg demographic, clinical, social) and information on exposures and potential confounders | N/A |
| | | (b) Indicate number of participants with missing data for each variable of interest | N/A |
| | | (c) *Cohort study*—Summarise follow-up time (eg, average and total amount) | N/A |
| Outcome data | 15* | *Cohort study*—Report numbers of outcome events or summary measures over time | *5* |
| | | *Case-control study*—Report numbers in each exposure category, or summary measures of exposure | |
| | | *Cross-sectional study*—Report numbers of outcome events or summary measures | |
| Main results | 16 | (*a*) Give unadjusted estimates and, if applicable, confounder-adjusted estimates and their precision (eg, 95% | 5-7 |

For peer review only

| | | | |
|---|---|---|---|
| | | confidence interval). Make clear which confounders were adjusted for and why they were included | |
| | | (*b*) Report category boundaries when continuous variables were categorized | |
| | | (*c*) If relevant, consider translating estimates of relative risk into absolute risk for a meaningful time period | |
| Other analyses | 17 | Report other analyses done—eg analyses of subgroups and interactions, and sensitivity analyses | N/A |
| **Discussion** | | | |
| Key results | 18 | Summarise key results with reference to study objectives | 7 |
| Limitations | 19 | Discuss limitations of the study, taking into account sources of potential bias or imprecision. Discuss both direction and magnitude of any potential bias | 8 |
| Interpretation | 20 | Give a cautious overall interpretation of results considering objectives, limitations, multiplicity of analyses, results from similar studies, and other relevant evidence | 7-8 |
| Generalisability | 21 | Discuss the generalisability (external validity) of the study results | 8 |
| **Other information** | | | |
| Funding | 22 | Give the source of funding and the role of the funders for the present study and, if applicable, for the original study on which the present article is based | 12 |

For peer review only

BMJ Open

# Trends in healthcare use in children aged less than 15 years; a population-based cohort study in England from 2007 to 2017

| | |
|---|---|
| Journal: | *BMJ Open* |
| Manuscript ID | bmjopen-2019-033761.R1 |
| Article Type: | Original research |
| Date Submitted by the Author: | 03-Jan-2020 |
| Complete List of Authors: | Ruzangi, Judith; Imperial College London,<br>Blair, Mitch; Imperial College London, Paediatrics<br>Cecil, E; Imperial College London, Primary Care and Public Health<br>Greenfield, Geva; Imperial College London, Department of Primary Care & Public Health<br>Bottle, Alex; Imperial College, Primary Care and Social Medicine<br>Hargreaves, Dougal; Imperial College London, Primary Care and Public Health<br>Saxena, Sonia; Imperial College London, School of Public Health; |
| <b>Primary Subject Heading</b>: | Health services research |
| Secondary Subject Heading: | General practice / Family practice, Health services research, Paediatrics |
| Keywords: | Healthcare use, GP consultations, Emergency department visits, Emergency admissions, Children |
| | |

SCHOLARONE™
Manuscripts

*I, the Submitting Author has the right to grant and does grant on behalf of all authors of the Work (as defined in the below author licence), an exclusive licence and/or a non-exclusive licence for contributions from authors who are: i) UK Crown employees; ii) where BMJ has agreed a CC-BY licence shall apply, and/or iii) in accordance with the terms applicable for US Federal Government officers or employees acting as part of their official duties; on a worldwide, perpetual, irrevocable, royalty-free basis to BMJ Publishing Group Ltd ("BMJ") its licensees and where the relevant Journal is co-owned by BMJ to the co-owners of the Journal, to publish the Work in this journal and any other BMJ products and to exploit all rights, as set out in our licence.*

*The Submitting Author accepts and understands that any supply made under these terms is made by BMJ to the Submitting Author unless you are acting as an employee on behalf of your employer or a postgraduate student of an affiliated institution which is paying any applicable article publishing charge ("APC") for Open Access articles. Where the Submitting Author wishes to make the Work available on an Open Access basis (and intends to pay the relevant APC), the terms of reuse of such Open Access shall be governed by a Creative Commons licence – details of these licences and which Creative Commons licence will apply to this Work are set out in our licence referred to above.*

*Other than as permitted in any relevant BMJ Author's Self Archiving Policies, I confirm this Work has not been accepted for publication elsewhere, is not being considered for publication elsewhere and does not duplicate material already published. I confirm all authors consent to publication of this Work and authorise the granting of this licence.*

For peer review only

# Trends in healthcare use in children aged less than 15 years; a population-based cohort study in England from 2007 to 2017

Judith Ruzangi,[1]* Mitch Blair,[2] Elizabeth Cecil, [1] Geva Greenfield, [1] Alex Bottle, [1] Dougal Hargreaves, [1] Sonia Saxena, [1]

Department of Primary Care & Public Health, School of Public Health, Imperial College London

***Corresponding author**: Judith Ruzangi;
Department of Primary Care and Public Health, School of Public Health, Imperial College London, 319 The Reynolds Building, St Dunstan's Road, London W6 8RP j.ruzangi@imperial.ac.uk

## Institutional affiliations

1. Department of Primary Care and Public Health, School of Public Health, Imperial College London. London. UK
2. River Island Academic Centre for Paediatrics and Child Health Imperial College London. London. UK Northwick Park Hospital Campus

## Guarantor

Professor Sonia Saxena: ORCID 0000-0003-3787-2083

Manuscript word count: 3387

For peer review only

# Abstract

## Objective

To describe changing use of primary care in relation to use of urgent care and planned hospital services by children aged less than 15 years in England in the decade following major primary care reforms from 2007-17

## Design

Population based retrospective cohort study.

## Methods

We used linked data from the Clinical Practice Research Datalink to study children's primary care consultations and use of hospital care including emergency department visits, emergency and elective admissions to hospital and outpatient visits to specialists.

## Results

Between 1 April 2007 to 31 March 2017 there were 7,604,024 GP consultations, 981,684 ED visits, 287,719 emergency hospital admissions, 2,253,533 outpatient visits and 194,034 elective admissions among 1,484,455 children aged less than 15 years. Age standardised GP consultation rates fell (-1.0% /year) to 1864 per 1000 child-years in 2017 in all age bands except infants rising by 1%/ year to 6722 per 1000/ child-years in 2017. ED visit rates increased by 1.6%/ year to 369 per 1000 child-years in 2017, with steeper rises of 3.9%/year in infants (780 per 1000 child-years in 2017). Emergency hospital admission rates rose steadily by 3%/ year to 86 per 1000 child-years and outpatient visit rates rose to 724 per 1000 child-years in 2017.

## Conclusions

Over the past decade since NHS primary care reforms, GP consultation rates have fallen for all children, except for infants. Children's use of hospital urgent and outpatient care has risen in all ages, especially infants. These changes signify the need for better access and provision of specialist and community-based support for families with young children.


Keywords: Healthcare use, Children, GP consultations, Emergency department visits, Emergency admissions

For peer review only

## Strengths and Limitations

- This is the largest nationally representative population-based study to date examining trends in children's healthcare use in the National Health Service in England
- The cohort design using individual level linked data from primary care and hospitals enabled a whole systems analysis of children's health care use
- Previous studies focused on children's primary care use have not previously been reported
- Improvements in data quality have enabled analysis of children's emergency department visit and outpatient visits
- The study population was children registered with a general practice, which underrepresents those from poorer postcodes, mobile or homeless populations

For peer review only

## Background

Primary care is at the heart of effective health systems but globally both primary care and hospital services are increasingly under pressure (1). The strengths of primary care include comprehensive, coordinated, preventive and planned care that has been shown to improve population health and reduce avoidable health system waste (2). In the United Kingdom (UK) National Health Service (NHS), over 98% of children are registered from birth with a family physician (known as a general practitioner (GP)) and receive preventive care including vaccinations and development checks as well as first contact care for acute problems (3). Infants and preschool children have among the highest visit rates of any age group to primary care (1). GPs also diagnose and manage chronic illnesses that emerge across the life course and play a crucial role in prioritising onwards referral for specialist care in hospital and outpatient settings. This cradle to grave model has been in place since the creation of the NHS over 70 years ago. However, major reforms in NHS primary care over the past decade are thought to have pushed clinical and administrative GP workloads to saturation point and reduced children's access to GPs in and out of hours (4–6). From 2004, most GPs opted out of providing out of hour's services and took on a greater role in active monitoring and management of a rising population burden of chronic conditions in adults, driven by financial incentive schemes (Table 1) (7). Face to face GP consulting rates increased by 10% and telephone consulting rates doubled in the decade to 2013/14 (1).

There is some evidence that children's use of hospital emergency services is sensitive to access and availability of primary care. In the period following primary care reforms of 2004, there were large increases in children's visits to emergency departments (ED) and rises in short stay hospital admissions for primary care sensitive conditions (7). A third of all British children aged less than 15 years currently visit ED each year (8). This figure rises to 40% in practices where access is poor (3). Two-thirds of children visit outside of normal GP consulting hours although the peak time for ED visits is between 4 and 6pm (3). A relatively high proportion of children visiting ED (15-80%) are discharged with no treatment or present with conditions that could potentially have been treated in primary care (9,10). Previous ecological studies have been unable to provide population-based estimates of children's health service use using individual level data across the primary and secondary care interface. The aim of this study was to describe trends in use of primary care in relation to use of urgent care and planned NHS hospital services by children aged less than 15 years in England in the decade following major primary care reforms.

Table 1 Summary of Major UK National health Service Reforms 2004 and key definitions

| |
|---|
| **Primary Care Reforms** (4)<br><br>Allowed primary care physicians (General Practitioners) greater flexibility in the services they provided, for example, opting out of responsibility for out- of-hours care.<br><br>Financial incentive scheme for primary care physicians to deliver clinical and organizational care, assessed through performance target achievement (the Quality and Outcomes Framework). |
| **Emergency care reforms**<br><br>Tightening of targets so that 98% of patients wait no more than 4 hours in an emergency department from arrival to hospital admission, transfer, or discharge (11). |
| **Hospital Reforms**<br><br>Introduction of payment-by-results schemes changed the way a hospital was paid, from a block contract payment system for service provision to one remunerating activity, such as episodes of care (12). |
| **General Practitioner consultation -** a consultation with a local family physician who delivers preventive care including vaccinations and development checks as well as first contact care for acute problems |

For peer review only

| | |
|---|---|
| **Outpatient visit –** a planned appointment with a hospital specialist (for example, a general paediatrician or a subspecialist such as a paediatric neurologist or paediatric gastroenterologist) | |
| **Urgent care –** unplanned immediate care received in emergency departments including urgent care centres that are commonly co-located within English EDs. | |

# Methods

## Study design and data sources

We carried out a cohort study using prospectively collected data from the Clinical Practice Research Datalink (CPRD), which is the largest validated primary care research database within the UK (13). It contains longitudinal, patient-level, anonymised computerised health records from more than 600 participating UK general practices (GPs) for 22 million patients (13). While only representing 8% of the UK population, age, sex, and ethnicity reflect demographics of the UK (13,14). We used CPRD linked to Hospital Episodes Statistics (HES) to obtain information on ED visits, emergency hospital admissions, elective admissions and outpatient attendance. HES contain information on NHS hospital activity in England. Ethical approval for this study was obtained from the Independent Scientific Advisory Committee (ISAC), which oversees research involving CPRD data (Protocol 18_139).

## Population

We constructed an open cohort including all children aged less than 15 years in HES linked CPRD registered "up to standard" CPRD-participating general practices during the study period from 01/04/2007 to 31/07/2017. Each child contributed to time of observation from birth or first registration date. Follow up for each year was continuous for each child until either the date they transferred out of practice, reached the age of 15 years, died or reached the end of the study period (31/07/2017), whichever came first.

Children were assigned to one of 4 developmental age groups; infants aged < 1-year, preschool children aged 1–4 years, school children aged 5–9 years and teenagers aged 10-14 years. Since CPRD data does not contain birth dates, we ascribed a birth date of the 1st of the recorded month and year of birth to each child. For children whose record had missing data for the month of their birth, we assumed a birthdate of the 1st Jan on their year of birth. Children contributed data on health service use at different ages across each year of the study period. For each financial year, age for each child was defined as the number of years since the birthdate of that child to the end of that financial year (31 March). Hence, this was an open cohort with children entering and leaving the sample as well as changes in age across the decade.

## Outcomes

Our main outcomes were age standardised rates for GP consultation, ED visits, emergency and elective hospital admissions and outpatient visits. We defined a GP consultation as any face to face consultation for illness that took place on practice premises, excluding consultations for routine preventive care (immunisation and development checks). An ED visit was defined as an emergency department attendance at a consultant-led emergency department with 24-hour service, full resuscitation facilities and designated accommodation for the reception of accident & emergency (A&E) patients and into other types of A&E/minor injury department. We excluded visits to consultant-led mono specialty A&E service such as specialist emergency eye units and NHS walk-in centres. Emergency admissions were admissions that were 'unpredictable and occur at short notice because of clinical need', whereas elective admissions were defined as occurring when the 'decision to admit can be separated in time from the actual admission'. We defined an outpatient attendance where a

For peer review only

child was recorded as having been seen by the intended care professional on the date of appointment on the HES outpatient appointments dataset (15). These outcomes are described in Supplementary File 1.

## Analysis

We calculated annual rates per 1000 child-years for each outcome for each financial year by summing the total number of events divided by the total child years of observation for all children aged less than 15 years. To enable comparison across years, rates were age standardised to the 2016 mid-year English population aged<15 years (16). Similarly, annual rates were calculated for each developmental age-group from 2007 to 2017. However, due to poor HES A&E data quality before 2011, we only report ED visit rates from 2011/2012 (17). We calculated as the change from baseline rate in 2007/8 to 2016/17, except for ED visits where we used a baseline reference year of 2011/12. We calculated the proportion of children with an ED visit who self-referred. We calculated the conversion proportion for emergency admission as the proportion of ED visits that led to emergency admissions.

## Patient and public involvement

Patients and the public were not directly involved in writing this paper.

# Results

Overall, 1,484,455 children from 408 practices contributed to this 10-year cohort study.
From 1 April 2007 to 31 March 2017, there were 7,604,024 GP consultations, 981,684 ED visits, 287,719 emergency admissions, 194,034 elective admissions, 2,253,533 outpatient visits among children aged less than 15 years in our population.

## GP consultations

The age standardised GP consultation rate in children less than 15 years dropped from 2005 to 1864 per 1000 child-years between 2007/8 and 2016/17 (Figure 1). In 2016/17, the telephone contact rates were 0.1 per child and home visits 1 per 1000 children The GP consultation rate in infants increased by 1%/ year from 6084 per 1000 child-years in 2007/2008 to 6722 per 1000 child-years in 2016/2017. The GP consultation rate in pre-school children and those aged 5-9 years fell from 2707 to 2368 per 1000 child-years and 1416 to 1217 per 1000 child-years in the same study period respectively. For children aged above 10 years, the rate was stable throughout the decade (1100 per 1000 child-years) (Table 2).

For peer review only

**Table 2 Annual rates of healthcare use per 1000 child-years and relative change from baseline 2007-2017[1,2]**

| | Infants < 1 years | | | Preschool children 1-4 years old | | | School children 5-9 years old | | | Teenagers 10-14 years old | | |
|---|---|---|---|---|---|---|---|---|---|---|---|---|
| | **2007/08** | **2016/17** | **% change[1] (95% CI)** | **2007/08** | **2016/17** | **% change[1] (95% CI)** | **2007/08** | **2016/17** | **% change[1] (95% CI)** | **2007/08** | **2016/17** | **% change[1] (95% CI)** |
| **General practice consultations** | 6084 | 6722 | 10.5 (8.9, 12.0) | 2707 | 2368 | -12.5 (-13.2, -11.8) | 1416 | 1218 | -14.0 (-14.8, -13.2) | 1143 | 1074 | -6.0 (-7.0, -5.0) |
| **Emergency admissions** | 312 | 410 | 31.6 (24.2, 39.1) | 109 | 123 | 12.8 (8.8, 16.8) | 38 | 39 | 2.8 (-2.9, 8.5) | 34 | 34 | -1.4 (-8.1, 5.2) |
| **Elective admissions** | 47 | 68 | 46.4 (14.1, 78.6) | 53 | 52 | -3.0 (-13.7, 7.6) | 48 | 47 | -2.4 (-10.3, 5.5) | 38 | 46 | 20.7 (-1.0, 42.3) |
| **Outpatient visits** | 883 | 1529 | 73.1 (66.0, 80.2) | 518 | 706 | 36.3 (33.7, 38.9) | 477 | 649 | 36.2 (33.8, 38.6) | 451 | 648 | 43.9 (41.1, 46.7) |
| | **2011/12** | **2016/17** | **% change (95% CI)** | **2011/12** | **2016/2017** | **% change (95% CI)** | **2011/12** | **2016/17** | **% change (95% CI)** | **2011/12** | **2016/17** | **% change (95% CI)** |
| **Emergency department visits[2]** | 562 | 780 | 38.6 (32.8, 44.5) | 419 | 497 | 18.8 (17.0, 20.5) | 226 | 249 | 10.3 (8.2,12.3) | 280 | 297 | 6.2 (4.2, 8.1) |

1. % change calculated as (rate in 2016/2017 – rate in 2007/2008)/rate in 2007/2008; 95% confidence interval (CI)
2. % change in emergency department visits calculated from baseline year starts from 2011/2012 due to poor data quality poor prior to this time

For peer review

### Urgent and emergency care; ED visits and emergency hospital admissions

From 2011 to 2017, the age standardised ED visit rate in children aged less than 15 years increased by 1.6%/ year from 319 to 369 per 1000 child-years. There was a much larger increase of 3.9%/ year in annual ED visit rate for infants compared with older children (562 to 780 per 1000 child-years from 2011 to 2017). Since 2011, every year, 70% of children visiting ED self-referred and 22% of ED visits led to an emergency admission. From 2007/2008 to 2016/2017, the age standardised emergency admission rate in children less than 15 years increased by 1.5%/ year from 75 to 86 per 1000 child-years and for infants by 3%/ year from 312 to 410 per 1000 child-years. However, the emergency admission rate in pre-school children and those aged above 5 years was stable throughout the decade (100-130 and <50 per 1000 child-years respectively).

### Planned care; outpatient visits and elective admissions

From 2007/8 to 2016/17, the annual age standardised outpatient visit rate in children aged less than 15 years increased from 508 to 724 per 1000 child-years (Figure 1). The outpatient visit rate in infants increased by 7.3%/ year from 883 to 1529 per 1000 child-years over the study period. Rates in pre-school children and those aged above 5 years also increased steadily throughout the same period from 500 to 700 per 1000 child-years (Figure 2). Age standardised elective admission rates in children less than 15 years over the same study period were stable from 46 to 49 per 1000 child-years but increased by 4.6%/ year from 47 to 68 per 1000 child-years for infants and by 2%/ year for teenagers from 38 to 46 per 1000 child-years. Rates were stable for pre-schoolers and school children (~50 per 1000 child-years).

## Discussion

### Key findings

Infant contact rates with GPs have increased year on year over the past decade following major reforms in UK primary care but older children are using primary care less. GP consultations are the major point of contact with healthcare services across all age groups. Children's use of urgent care in hospitals, including visits to EDs and emergency admission rates, has increased particularly among infants. There have been sharp increases in planned care including outpatient activity and admissions for elective care in infants and teenage children.

### Comparison with previous studies

We have previously reported GP consultation rates for illness in children of a similar magnitude for a CPRD cohort born in 2000 and another study of healthcare use in primary care study that are remarkably consistent with our findings here for children (1,18). Infants, however, seem to be seeing their GP more frequently in 2017 compared with a decade ago. The expansion in the use of urgent care is consistent with trends in the UK and other high-income country settings where emergency admission rates in both children and adults have been rising for several years (7,19). There is little research on children's outpatient service use and elective admissions but are consistent with overall rises in the total number of elective admissions, referrals to outpatient services and total outpatient attendances in English hospitals, which has increased markedly since 2007 (20–22).

Rising needs in the child population and demand from families are most likely contributors to the patterns of overall increasing healthcare use by children seen here (8). Preterm birth is increasing and is the single biggest cause of neonatal morbidity and mortality in the UK and other countries where survival rates are increasing (23). Thus, rising emergency admissions in infants could be explained by a growing morbidity burden in infants compared with other children. Recurrent admissions in children and young people with chronic conditions contribute substantially to total emergency admissions (24). A report projecting the State of Child Health in 2030 concluded that Britain's children who make up

For peer review only

nearly 20% of the population have a rising health burden and poorer health compared with other wealthy countries. Even more concerning is a widening in the gap in health between wealthy and poor children (25). Hence growing morbidity burden particularly in deprived groups may be contributing to the rise in health care use.

Health system failures to provide responsive care in the community to meet the needs of the children and families might well explain the rise in urgent care use. Parents prefer using their regular general GP when their child is unwell, but they choose to visit the ED if they perceive their child's condition as serious or cannot access primary care (26–28). We found the majority (70%) of visits were self-referred and 80% of children discharged after their ED visit. It is estimated that 10% of infants attending the ED have no underlying medical problem (29), yet this does not mean that 90% need to seek emergency care if they require support to care for their child. The 2004 primary care healthcare reforms focused heavily on improving chronic disease management in adults with long term conditions and allowed primary care physicians to opt out of providing acute primary care services during evenings and weekends. This reduced access that may explain rising emergency department visits (3) . The introduction of four-hour waiting target in ED in 2004 has been previously suggested as a contributing factor to increases in emergency admission rates (6,30). However, less than 5% of children's admission breach the 4-hour waiting time target so pressures within EDs are unlikely to explain the rising admission rate (31). Similarly, there is little evidence that ED staff have lowered their threshold for admitting children in our findings since the attendance admission ratio remained around 1 in 5 across the decade. Thus, our findings and previous studies point to mounting pressures in the community and illustrate the remarkable ability of hospitals in England to respond to the needs of families with young children.

## Strengths and Limitations

Our study is the first to report on trends in healthcare use by children for both primary and hospital care in the UK. This is among the largest studies to report healthcare use in children across the primary and secondary care interface in a health system with universal health coverage. The strengths of this study include its size, national coverage and representativeness of the UK population of both CPRD and HES data sources widely used for research. This reduces the possibility that our findings are due to chance. Few countries have health systems that enable the study of individual level data on children and links between primary care and hospital data.
However, there are several limitations to consider. First, in common with many observational studies using administrative records, data quality is variable. Our datasets did not include consultations in some urgent care settings including NHS walk in centres private providers. Use of HES A&E data was initiated in 2007/2008 but data quality and coverage did not improve until 2009. Hence we restricted our analysis of EDs to the period from 2011 onwards (17). HES Outpatient data were first available in 2003/2004 and have been validated for research from 2015/16.The rate of outpatient attendances in 2015/2016 has been ascribed to fluctuation in data quality and thus has not been reported in this paper (22). Second, we used a proxy for birthdate as CPRD only provides month of birth for children aged less than 15 years. This would overestimate the follow up period and underestimate the rate. Third, children living in deprived areas are more likely to deregister from CPRD practices than children residing in more affluent postcodes. Hence, our findings may not be generalisable to these groups (32). Finally, our findings may not be generalisable to certain patient groups missing from CPRD such as children using private health care services and homeless children (13).

## Implications and future research

The large sustained rises we report in primary care, urgent and planned care in hospital in infants have important implications for primary care. If rising urgent care use and falling contact rates with general practitioners are evidence of difficulties in children's access to GP appointments this is an

For peer review only

important area for reform. It brings into question the continued selective financial incentive schemes for general practitioners to manage a rising multimorbidity burden in older patients. It is of concern that general practitioners are not universally trained in paediatrics and child health medicine as has been the recommendation of the European Academy of Paediatrics recently (33). Regardless, upwards trends in planned and urgent healthcare use by children have major resource and cost implications for the NHS and we recommend the drivers and mediators of these changes warrant further research (3,34,35) . A further breakdown of data, which includes ethnic background, index of multiple deprivation, repeat attendance, presenting problems and case mix would be immensely helpful in understanding these trends. The number of health visitors and midwives who are providing support has decreased in England and Wales in recent years as a result of funding cuts (36). Accurate data on the effectiveness of this workforce in supporting parents is currently lacking but is recommended as an area for future research. Finally, there is scope to explore innovative approaches including parenting programmes and peer support for increasing parent's confidence and capacity in caring for their children.

## Conclusions

Over the past decade in England, general practice consultations have fallen for all children, except for infants. Children's use of hospital urgent and outpatient care has risen in all ages, especially infants. These changes may signify the need for better access to primary care and provision of specialist and community-based support for families with young children. Research to understand drivers and identify solutions to rising health service use is urgently needed.

For peer review only

## References

1. Hobbs FDR, Bankhead C, Mukhtar T, Stevens S, Perera-Salazar R, Holt T, et al. Clinical workload in UK primary care: a retrospective analysis of 100 million consultations in England, 2007–14. Lancet. 2016;387(10035):2323–30.
2. Shi Leiyu MJSB. Contribution of primary care to health systems and health. Milbank Q. 2005 Sep 1;83(3):457–502.
3. Cecil E, Bottle A, Cowling TE, Majeed A, Wolfe I, Saxena S. Primary Care Access, Emergency Department Visits, and Unplanned Short Hospitalizations in the UK. Pediatrics. 2016 Feb 1;137(2):e20151492.
4. Investing in General Practice The New General Medical Services Contract.
5. Roland M, Campbell S. Successes and Failures of Pay for Performance in the United Kingdom. Hamel MB, editor. N Engl J Med. 2014 May 15;370(20):1944–9.
6. Gill PJ, Goldacre MJ, Mant D, Heneghan C, Thomson A, Seagroatt V, et al. Increase in emergency admissions to hospital for children aged under 15 in England, 1999–2010: national database analysis. Arch Dis Child. 2013 May 1;98(5):328–34.
7. Cecil E, Bottle A, Sharland M, Saxena S. Impact of UK Primary Care Policy Reforms on Short-Stay Unplanned Hospital Admissions for Children With Primary Care–Sensitive Conditions. Ann Fam Med. 2015;13(3):214.
8. Keeble E, Kossarova L. Focus on: Emergency hospital care for children and young people. 2017.
9. Smith L, Narang Y, Ibarz Pavon AB, Edwardson K, Bowers S, Jones K, et al. To GP or not to GP: a natural experiment in children triaged to see a GP in a tertiary paediatric emergency department (ED). BMJ Qual Saf. 2017 Sep;bmjqs-2017-006605.
10. Gnani S, Morton S, Ramzan F, Davison M, Ladbrooke T, Majeed A, et al. Healthcare use among preschool children attending GP-led urgent care centres: a descriptive, observational study. BMJ Open. 2016 Jun;6(6):e010672.
11. The NHS plan : a plan for investment : a plan for reform : presented to Parliament by the Secretary of State for Health by command of Her Majesty : July 2000.
12. [ARCHIVED CONTENT] Improving coding, costing and commissioning - Audit Commission [Internet]. [cited 2019 Dec 13]. Available from: https://webarchive.nationalarchives.gov.uk/20111202180943/http://www.audit-commission.gov.uk/health/nationalstudies/pbr/pbr2011/Pages/pbr2011_copy.aspx#downloads
13. Herrett E, Gallagher AM, Bhaskaran K, Forbes H, Mathur R, Van Staa T, et al. Data Resource Profile: Clinical Practice Research Datalink (CPRD).
14. Mathur R, Bhaskaran K, Chaturvedi N, Leon DA, Van Staa T, Grundy E, et al. Completeness and usability of ethnicity data in UK-based primary care and hospital databases. J Public Heal (United Kingdom). 2014;36(4):684–92.
15. Hospital Episode Statistics Data Dictionary - NHS Digital [Internet]. [cited 2019 May 31].
16. Population estimates for the UK, England and Wales, Scotland and Northern Ireland - Office for National Statistics [Internet]. [cited 2019 Jun 7].
17. HES 2011-12 A&E Data Quality Note Experimental data set. 2013.
18. Cecil E, Bottle A, Ma R, Hargreaves DS, Wolfe I, Mainous AG, et al. Impact of preventive primary care on children's unplanned hospital admissions: a population-based birth cohort study of UK children 2000–2013. BMC Med. 2018 Dec 17;16(1):151.
19. Health at a Glance 2015: OECD Indicators. Paris: OECD Publishing; 2015.
20. Baker C. NHS Key Statistics: England, May 2019.
21. Dreyer K, Williamson RAP, Hargreaves DS, Rosen R, Deeny SR. Associations between parental mental health and other family factors and healthcare utilisation among children and young people: a retrospective, cross-sectional study of linked healthcare data. BMJ Paediatr Open. 2018 Jul 30;2(1):e000266.
22. Hospital Outpatient Activity - NHS Digital [Internet]. [cited 2019 May 31].
23. Blencowe H, Cousens S, Oestergaard MZ, Chou D, Moller A-B, Narwal R, et al. National, regional, and worldwide estimates of preterm birth rates in the year 2010 with time trends since 1990 for selected countries: a systematic analysis and implications. Lancet (London, England). 2012 Jun 9;379(9832):2162–72.
24. Wijlaars LP, Hardelid P, Woodman J, Allister J, Cheung R, Gilbert R. Contribution of recurrent admissions in children and young people to emergency hospital admissions: retrospective cohort analysis of hospital episode statistics. Arch Dis Child. 2015 Sep;100(9):845–9.
25. Viner R, Ward J, Cheung R, Wolfe I, Hargreaves D. Child health in 2030 in England:

For peer review only

comparisons with other wealthy countries.
26. Williams A, O'Rourke P, Keogh S. Making choices: why parents present to the emergency department for non-urgent care. Arch Dis Child. 2009 Oct 1;94(10):817–20.
27. Woolfenden S, Ritchie J, Hanson R, Nossar V. Parental use of a paediatric emergency department as an ambulatory care service. Aust N Z J Public Health. 2000 Apr 1;24(2):204–6.
28. Maguire S, Ranmal R, Komulainen S, Pearse S, Maconochie I, Lakhanpaul M, et al. Which urgent care services do febrile children use and why? Arch Dis Child. 2011 Sep 1;96(9):810–6.
29. Heys M, Kwong H-M, Reed J, Blair M. What do we really know about infants who attend Accident and Emergency departments? Perspect Public Health. 2014 Mar;134(2):93–100.
30. Saxena S, Bottle A, Gilbert R, Sharland M. Increasing Short-Stay Unplanned Hospital Admissions among Children in England; Time Trends Analysis '97–'06. van Baal PHM, editor. PLoS One. 2009 Oct;4(10):e7484.
31. Blunt I. Focus on : A & E attendances.Why are patients waiting longer. 2014.
32. Howe LD, Tilling K, Galobardes B, Lawlor DA. Loss to follow-up in cohort studies: bias in estimates of socioeconomic inequalities. Epidemiology. 2013 Jan;24(1):1–9.
33. Završnik J, Stiris T, Schrier L, Russell RR, del Torso S, Valiulis A, et al. Basic training requirements for health care professionals who care for children. Eur J Pediatr. 2018 Sep 25;177(9):1413–7.
34. Watson M, Blair M. Emergency departments and minor illness: Some behavioural insights. Archives of Disease in Childhood. 2018;103(4):309–10.
35. Shraim M, Blagojevic-Bucknall M, Mallen CD, Dunn KM. Repeated primary care consultations for non-specific physical symptoms in children in UK: a cohort study. BMC Fam Pract. 2014 Dec 5;15:195.
36. Buchan J, Charlesworth A, Gershlick B, Seccombe I. A critical moment: NHS staffing trends, retention and attrition. 2019.

For peer review only

# Additional Information

## Contributors:

SS and MB conceived and designed this study. JR prepared the data and carried out the statistical analysis overseen by EC and AB. JR, MB, EC, GG, AB, DH and SS took part in interpreting the data for this study, commented on and helped to revise drafts of this paper and have approved the final version. SS is the guarantor.

**Funding:** This article presents independent research supported by the National Institute for Health Research (NIHR) School for Public Health research (SS) the Collaborations for Leadership in Applied Health Research and Care (CLAHRC) programme for Northwest London (PD-SPH-2015-10055). The Dr Foster Unit at Imperial is affiliated with the National Institute of Health Research (NIHR) Imperial Patient Safety Translational Research Centre. The NIHR Imperial Patient Safety Translational Centre is a partnership between the Imperial College Healthcare NHS Trust and Imperial College London. The Dr Foster Unit at Imperial College are grateful for support from the NIHR Biomedical Research Centre funding scheme.

**Disclaimer:** The views expressed are those of the authors and not necessarily those of the NHS, the NIHR or the Department of Health and Social Care.

**Competing interests:** The authors declare that they have no competing interests.

**Patient consent:** Not required.

**Ethics approval:** We obtained ethical and scientific approval for the use of CPRD for our study from the Independent Scientific Advisory Committee Protocol number: 18_139.

**Provenance and peer review:** Not commissioned; externally peer reviewed.

**Data sharing Copyright information:** due to data sharing agreements, data are unavailable for sharing publicly.

**Copyright information:** This is an open access article distributed in accordance with the Creative Commons Attribution Non Commercial (CC BY-NC 4.0) license, which permits others to distribute, remix, adapt, build upon this work non-commercially, and license their derivative works on different terms, provided the original work is properly cited, appropriate credit is given, any changes made indicated, and the use is non-commercial. See: http://creativecommons.org/licenses/by-nc/4.0/.

**Figure legends**

Figure 1 Healthcare use in children aged less than 15 years

Figure 2 Healthcare use in children by developmental age groups

For peer review only

For peer review only

Figure 1 Healthcare use in children aged less than 15 years

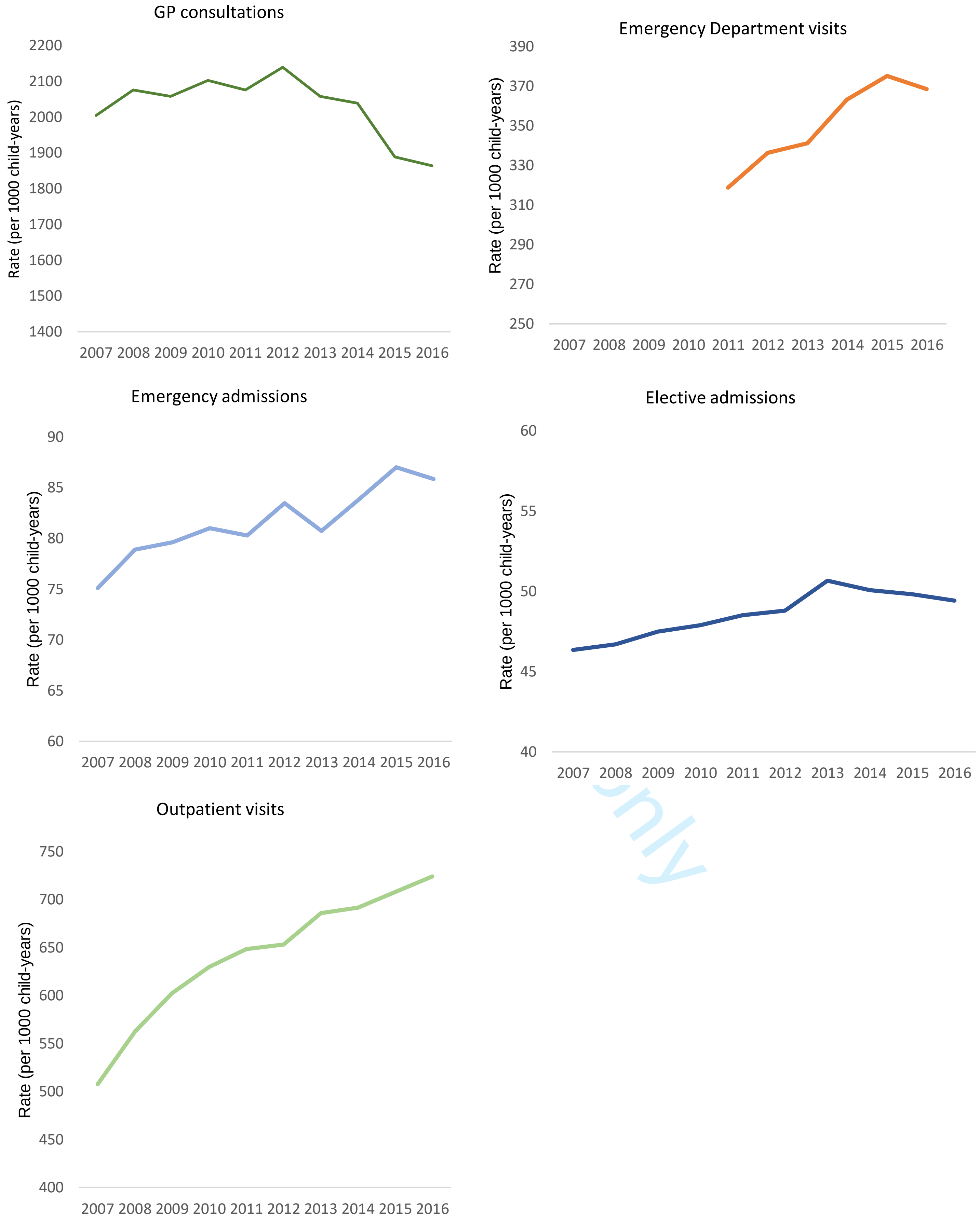


Rates were directly age-standardised to the 2016 mid-year English population

For peer review only - http://bmjopen.bmj.com/site/about/guidelines.xhtml

Figure 2 Healthcare use in children by developmental age groups

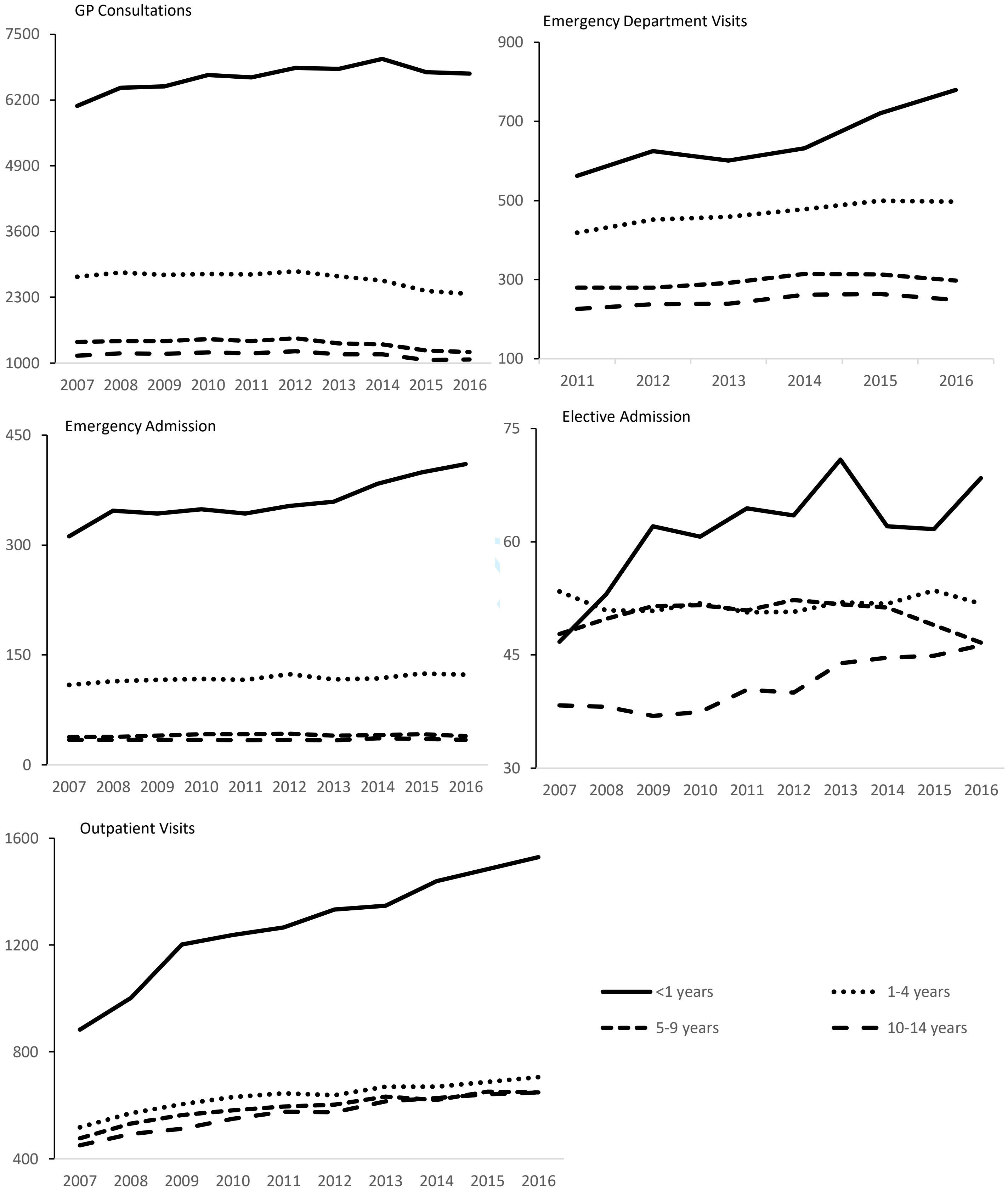

# Supplementary File 1

### Creating GP consultations

We defined a GP consultation as any face to face consultation for illness that took place on practice premises, excluding consultations for routine preventive care (immunisation and development checks).

Using CPRD clinical data we:

1) Defined a GP consultation as direct contact between a GP and a child using the consultation type look up table COT if the event date was recorded. Direct consultations were for Clinic, Follow-up/routine visit, Surgery consultation, Acute visit, Emergency Consultation and Co-op Surgery Consultation. Thus, selected Constype = (1, 3, 9, 11, 18, 36).

2) Differentiated between consultation with a GP and consultation with another practice member of staff by highlighting the staff who were GPs using the staff id and the Role of staff look up table ROL. Roles classified as GP were Senior Partner, Partner, Assistant, Associate, Locum, GP Registrar, Salaried Partner and GP Retainer. Thus, selected Role code = (1, 2, 3, 4, 7, 8, 47, 50).

3) Excluded records that were administrative using the Consultation type look up table SED. Thus, excluded type = administration (6).

4) We excluded GP consultations for preventive care (using the patient id and consultation ids isolated in preventive care records below). Thus, excluded consultations for immunisations, child development check and routine child health check.

### Supplementary Table 1: Read Codes identifying preventive care consultations

| Description | V2 Read Codes |
|---|---|
| **Preventive care** | |
| Child development checks | 64… |
| Routine child health check | ZV202 |
| Vaccinations | All vaccinations (from CPRD immunization files) |

### Creating Emergency Department (ED) visits

An ED visit was defined as an emergency department attendance at a consultant-led emergency department with 24-hour service, full resuscitation facilities and designated accommodation for the reception of accident & emergency (A&E) patients and into other types of A&E/minor injury department. We excluded visits to consultant-led mono specialty A&E service such as specialist emergency eye units and NHS walk-in centres.

Using HES Accidents & Emergency data we:

1) Defined an ED visit using the field 'AEDEPTTYPE' if 'ARRIVALDATE' was recorded. Thus, selected 'AEDEPTTYPE' = (1,3)

2) Defined source of referral using the field 'AEREFSOURCE'. Thus, selected self-referrals = 'AEREFSOURCE' = (1)

For peer review only

**Creating Emergency and Elective admissions**
Emergency admissions are unpredictable and occur at short notice because of clinical need whereas elective admissions are defined as occurring when the decision to admit can be separated in time from the actual admission.

Using HES Admitted Patient Care data we:

1) Defined an admission using the method of admission field 'ADMIMETH' if 'ADMIDATE' was recorded.

2) Differentiated between emergency admissions and elective admissions using the method of admission field 'ADMIMETH' below.

**Supplementary Table 2: ADMIMETH codes identifying emergency and elecive admissions**

| Outcome | ADMIMETH code used |
|---|---|
| Emergency admissions | 21, 22, 23, 24, 25, 28, 2A, 2B, 2C, 2D |
| Elective admissions | 11, 12, 13 |

**Creating outpatient visits**
We defined an outpatient attendance where a child was recorded as having been seen by the intended care professional on the date of appointment on the HES outpatient appointments dataset.

Using HES Outpatients data we:

1) Defined an attendance to Outpatients using the attended or did not attend field 'ATTENDED' if the field 'APPTDATE' for the appointment date was recorded. Thus, selected 'ATTENDED' = (5, 6).

For peer review only

# Annex 1: STROBE Statement—checklist of items that should be included in reports of observational studies

| | Item No | Recommendation | Pg. |
|---|---|---|---|
| **Title and abstract** | 1 | (*a*) Indicate the study's design with a commonly used term in the title or the abstract | 1 |
| | | (*b*) Provide in the abstract an informative and balanced summary of what was done and what was found | 2 |
| **Introduction** | | | |
| Background/rationale | 2 | Explain the scientific background and rationale for the investigation being reported | 4 |
| Objectives | 3 | State specific objectives, including any prespecified hypotheses | 4 |
| **Methods** | | | |
| Study design | 4 | Present key elements of study design early in the paper | 4 |
| Setting | 5 | Describe the setting, locations, and relevant dates, including periods of recruitment, exposure, follow-up, and data collection | 4 |
| Participants | 6 | (*a*) *Cohort study*—Give the eligibility criteria, and the sources and methods of selection of participants. Describe methods of follow-up<br>*Case-control study*—Give the eligibility criteria, and the sources and methods of case ascertainment and control selection. Give the rationale for the choice of cases and controls | 4 |

For peer review only

| | | | |
|---|---|---|---|
| | | *Cross-sectional study*—Give the eligibility criteria, and the sources and methods of selection of participants | |
| | | (*b*) *Cohort study*—For matched studies, give matching criteria and number of exposed and unexposed<br>*Case-control study*—For matched studies, give matching criteria and the number of controls per case | |
| Variables | 7 | Clearly define all outcomes, exposures, predictors, potential confounders, and effect modifiers. Give diagnostic criteria, if applicable | 5 |
| Data sources/ measurement | 8* | For each variable of interest, give sources of data and details of methods of assessment (measurement). Describe comparability of assessment methods if there is more than one group | 5 |
| Bias | 9 | Describe any efforts to address potential sources of bias | 5 |
| Study size | 10 | Explain how the study size was arrived at | N/A |
| Quantitative variables | 11 | Explain how quantitative variables were handled in the analyses. If applicable, describe which groupings were chosen and why | 5 |
| Statistical methods | 12 | (*a*) Describe all statistical methods, including those used to control for confounding | 5 |
| | | (*b*) Describe any methods used to examine subgroups and interactions | N/A |
| | | (*c*) Explain how missing data were addressed | 5 |
| | | (*d*) *Cohort study*—If applicable, explain how loss to follow-up was addressed | N/A |

For peer review only

| | | | |
|---|---|---|---|
| | | *Case-control study*—If applicable, explain how matching of cases and controls was addressed<br>*Cross-sectional study*—If applicable, describe analytical methods taking account of sampling strategy | |
| | | (*e*) Describe any sensitivity analyses | N/A |

**Results**

| | | | |
|---|---|---|---|
| Participants | 13* | (a) Report numbers of individuals at each stage of study—eg numbers potentially eligible, examined for eligibility, confirmed eligible, included in the study, completing follow-up, and analysed | 5 |
| | | (b) Give reasons for non-participation at each stage | N/A |
| | | (c) Consider use of a flow diagram | |
| Descriptive data | 14* | (a) Give characteristics of study participants (eg demographic, clinical, social) and information on exposures and potential confounders | N/A |
| | | (b) Indicate number of participants with missing data for each variable of interest | N/A |
| | | (c) *Cohort study*—Summarise follow-up time (eg, average and total amount) | N/A |
| Outcome data | 15* | *Cohort study*—Report numbers of outcome events or summary measures over time | *5* |
| | | *Case-control study*—Report numbers in each exposure category, or summary measures of exposure | |
| | | *Cross-sectional study*—Report numbers of outcome events or summary measures | |
| Main results | 16 | (*a*) Give unadjusted estimates and, if applicable, confounder-adjusted estimates and their precision (eg, 95% | 5-7 |

For peer review only

| | | | |
|---|---|---|---|
| | | confidence interval). Make clear which confounders were adjusted for and why they were included | |
| | | (*b*) Report category boundaries when continuous variables were categorized | |
| | | (*c*) If relevant, consider translating estimates of relative risk into absolute risk for a meaningful time period | |
| Other analyses | 17 | Report other analyses done—eg analyses of subgroups and interactions, and sensitivity analyses | N/A |
| **Discussion** | | | |
| Key results | 18 | Summarise key results with reference to study objectives | 7 |
| Limitations | 19 | Discuss limitations of the study, taking into account sources of potential bias or imprecision. Discuss both direction and magnitude of any potential bias | 8 |
| Interpretation | 20 | Give a cautious overall interpretation of results considering objectives, limitations, multiplicity of analyses, results from similar studies, and other relevant evidence | 7-8 |
| Generalisability | 21 | Discuss the generalisability (external validity) of the study results | 8 |
| **Other information** | | | |
| Funding | 22 | Give the source of funding and the role of the funders for the present study and, if applicable, for the original study on which the present article is based | 12 |

For peer review only

BMJ Open

# Trends in healthcare use in children aged less than 15 years; a population-based cohort study in England from 2007 to 2017



| | |
|---|---|
| Journal: | *BMJ Open* |
| Manuscript ID | bmjopen-2019-033761.R2 |
| Article Type: | Original research |
| Date Submitted by the Author: | 16-Mar-2020 |
| Complete List of Authors: | Ruzangi, Judith; Imperial College London,<br>Blair, Mitch; Imperial College London, Paediatrics<br>Cecil, E; Imperial College London, Primary Care and Public Health<br>Greenfield, Geva; Imperial College London, Department of Primary Care & Public Health<br>Bottle, Alex; Imperial College, Primary Care and Social Medicine<br>Hargreaves, Dougal; Imperial College London, Primary Care and Public Health<br>Saxena, Sonia; Imperial College London, School of Public Health; |
| <b>Primary Subject Heading</b>: | Health services research |
| Secondary Subject Heading: | General practice / Family practice, Health services research, Paediatrics |
| Keywords: | Healthcare use, GP consultations, Emergency department visits, Emergency admissions, Children |
| | |



SCHOLARONE™
Manuscripts

*I, the Submitting Author has the right to grant and does grant on behalf of all authors of the Work (as defined in the below author licence), an exclusive licence and/or a non-exclusive licence for contributions from authors who are: i) UK Crown employees; ii) where BMJ has agreed a CC-BY licence shall apply, and/or iii) in accordance with the terms applicable for US Federal Government officers or employees acting as part of their official duties; on a worldwide, perpetual, irrevocable, royalty-free basis to BMJ Publishing Group Ltd ("BMJ") its licensees and where the relevant Journal is co-owned by BMJ to the co-owners of the Journal, to publish the Work in this journal and any other BMJ products and to exploit all rights, as set out in our licence.*

*The Submitting Author accepts and understands that any supply made under these terms is made by BMJ to the Submitting Author unless you are acting as an employee on behalf of your employer or a postgraduate student of an affiliated institution which is paying any applicable article publishing charge ("APC") for Open Access articles. Where the Submitting Author wishes to make the Work available on an Open Access basis (and intends to pay the relevant APC), the terms of reuse of such Open Access shall be governed by a Creative Commons licence – details of these licences and which Creative Commons licence will apply to this Work are set out in our licence referred to above.*

*Other than as permitted in any relevant BMJ Author's Self Archiving Policies, I confirm this Work has not been accepted for publication elsewhere, is not being considered for publication elsewhere and does not duplicate material already published. I confirm all authors consent to publication of this Work and authorise the granting of this licence.*

For peer review only

# Trends in healthcare use in children aged less than 15 years; a population-based cohort study in England from 2007 to 2017

Judith Ruzangi,[1]* Mitch Blair,[2] Elizabeth Cecil, [1] Geva Greenfield, [1] Alex Bottle, [1] Dougal Hargreaves, [1] Sonia Saxena, [1]

Department of Primary Care & Public Health, School of Public Health, Imperial College London

***Corresponding author**: Judith Ruzangi;
Department of Primary Care and Public Health, School of Public Health, Imperial College London, 319 The Reynolds Building, St Dunstan's Road, London W6 8RP j.ruzangi@imperial.ac.uk

## Institutional affiliations

1. Department of Primary Care and Public Health, School of Public Health, Imperial College London. London. UK
2. River Island Academic Centre for Paediatrics and Child Health Imperial College London. London. UK Northwick Park Hospital Campus

## Guarantor

Professor Sonia Saxena: ORCID 0000-0003-3787-2083

Manuscript word count: 3240

For peer review only

# Abstract

## Objective

To describe changing use of primary care in relation to use of urgent care and planned hospital services by children aged less than 15 years in England in the decade following major primary care reforms from 2007-17

## Design

Population based retrospective cohort study.

## Methods

We used linked data from the Clinical Practice Research Datalink to study children's primary care consultations and use of hospital care including emergency department visits, emergency and elective admissions to hospital and outpatient visits to specialists.

## Results

Between 1 April 2007 to 31 March 2017 there were 7,604,024 GP consultations, 981,684 ED visits, 287,719 emergency hospital admissions, 2,253,533 outpatient visits and 194,034 elective admissions among 1,484,455 children aged less than 15 years. Age standardised GP consultation rates fell (-1.0% /year) to 1864 per 1000 child-years in 2017 in all age bands except infants rising by 1%/ year to 6722 per 1000/ child-years in 2017. ED visit rates increased by 1.6%/ year to 369 per 1000 child-years in 2017, with steeper rises of 3.9%/year in infants (780 per 1000 child-years in 2017). Emergency hospital admission rates rose steadily by 3%/ year to 86 per 1000 child-years and outpatient visit rates rose to 724 per 1000 child-years in 2017.

## Conclusions

Over the past decade since NHS primary care reforms, GP consultation rates have fallen for all children, except for infants. Children's use of hospital urgent and outpatient care has risen in all ages, especially infants. These changes signify the need for better access and provision of specialist and community-based support for families with young children.


Keywords: Healthcare use, Children, GP consultations, Emergency department visits, Emergency admissions

For peer review only

## Strengths and Limitations

- This is the largest nationally representative population-based study to date examining trends in children's healthcare use in the National Health Service in England
- The cohort design using individual level linked data from primary care and hospitals enabled a whole systems analysis of children's health care use
- Previous studies focused on children's primary care use have not previously been reported
- Improvements in data quality have enabled analysis of children's emergency department visit and outpatient visits
- The study population was children registered with a general practice, which underrepresents those from poorer postcodes, mobile or homeless populations

For peer review only

## Background

Primary care is at the heart of effective health systems but globally both primary care and hospital services are increasingly under pressure (1). The strengths of primary care include comprehensive, coordinated, preventive and planned care that has been shown to improve population health and reduce avoidable health system waste (2). In the United Kingdom (UK) National Health Service (NHS), over 98% of children are registered from birth with a family physician (known as a general practitioner (GP)) and receive preventive care including vaccinations and development checks as well as first contact care for acute problems (3). Infants and preschool children have among the highest visit rates of any age group to primary care (1). GPs also diagnose and manage chronic illnesses that emerge across the life course and play a crucial role in prioritising onwards referral for specialist care in hospital and outpatient settings. This cradle to grave model has been in place since the creation of the NHS over 70 years ago. However, major reforms in NHS primary care over the past decade are thought to have pushed clinical and administrative GP workloads to saturation point and reduced children's access to GPs in and out of hours (4–6). From 2004, most GPs opted out of providing out of hour's services and took on a greater role in active monitoring and management of a rising population burden of chronic conditions in adults, driven by financial incentive schemes (Table 1) (7). Face to face GP consulting rates increased by 10% and telephone consulting rates doubled in the decade to 2013/14 (1).

There is some evidence that children's use of hospital emergency services is sensitive to access and availability of primary care. In the period following primary care reforms of 2004, there were large increases in children's visits to emergency departments (ED) and rises in short stay hospital admissions for primary care sensitive conditions (7). A third of all British children aged less than 15 years currently visit ED each year (8). This figure rises to 40% in practices where access is poor (3). Two-thirds of children visit outside of normal GP consulting hours although the peak time for ED visits is between 4 and 6pm (3). A relatively high proportion of children visiting ED (15-80%) are discharged with no treatment or present with conditions that could potentially have been treated in primary care (9,10). Previous ecological studies have been unable to provide population-based estimates of children's health service use using individual level data across the primary and secondary care interface. The aim of this study was to describe trends in use of primary care in relation to use of urgent care and planned NHS hospital services by children aged less than 15 years in England in the decade following major primary care reforms.

Table 1 Summary of Major UK National health Service Reforms 2004 and key definitions

| |
|---|
| **Primary Care Reforms** (4)<br><br>Allowed primary care physicians (General Practitioners) greater flexibility in the services they provided, for example, opting out of responsibility for out- of-hours care.<br><br>Financial incentive scheme for primary care physicians to deliver clinical and organizational care, assessed through performance target achievement (the Quality and Outcomes Framework). |
| **Emergency care reforms**<br><br>Tightening of targets so that 98% of patients wait no more than 4 hours in an emergency department from arrival to hospital admission, transfer, or discharge (11). |
| **Hospital Reforms**<br><br>Introduction of payment-by-results schemes changed the way a hospital was paid, from a block contract payment system for service provision to one remunerating activity, such as episodes of care (12). |
| **General Practitioner consultation -** a consultation with a local family physician who delivers preventive care including vaccinations and development checks as well as first contact care for acute problems |

| | |
|---|---|
| **Outpatient visit –** a planned appointment with a hospital specialist (for example, a general paediatrician or a subspecialist such as a paediatric neurologist or paediatric gastroenterologist) | |
| **Urgent care –** unplanned immediate care received in emergency departments including urgent care centres that are commonly co-located within English EDs. | |

# Methods

## Study design and data sources

We carried out a cohort study using prospectively collected data from the Clinical Practice Research Datalink (CPRD), which is the largest validated primary care research database within the UK (13). It contains longitudinal, patient-level, anonymised computerised health records from more than 600 participating UK general practices (GPs) for 22 million patients (13). While only representing 8% of the UK population, age, sex, and ethnicity reflect demographics of the UK (13,14). We used CPRD linked to Hospital Episodes Statistics (HES) to obtain information on ED visits, emergency hospital admissions, elective admissions and outpatient attendance. HES contain information on NHS hospital activity in England. Ethical approval for this study was obtained from the Independent Scientific Advisory Committee (ISAC), which oversees research involving CPRD data (Protocol 18_139).

## Population

We constructed an open cohort including all children aged less than 15 years in HES linked CPRD registered “up to standard” CPRD-participating general practices during the study period from 01/04/2007 to 31/07/2017. Each child contributed to time of observation from birth or first registration date. Follow up for each year was continuous for each child until either the date they transferred out of practice, reached the age of 15 years, died or reached the end of the study period (31/07/2017), whichever came first.

Children were assigned to one of 4 developmental age groups; infants aged < 1-year, preschool children aged 1–4 years, school children aged 5–9 years and teenagers aged 10-14 years. Since CPRD data does not contain birth dates, we ascribed a birth date of the 1st of the recorded month and year of birth to each child. For children whose record had missing data for the month of their birth, we assumed a birthdate of the 1st Jan on their year of birth. Children contributed data on health service use at different ages across each year of the study period. For each financial year, age for each child was defined as the number of years since the birthdate of that child to the end of that financial year (31 March). Hence, this was an open cohort with children entering and leaving the sample as well as changes in age across the decade.

## Outcomes

Our main outcomes were age standardised rates for GP consultation, ED visits, emergency and elective hospital admissions and outpatient visits. We defined a GP consultation as any face to face consultation for illness that took place on practice premises, excluding consultations for routine preventive care (immunisation and development checks). An ED visit was defined as an emergency department attendance at a consultant-led emergency department with 24-hour service, full resuscitation facilities and designated accommodation for the reception of accident & emergency (A&E) patients and into other types of A&E/minor injury department. We excluded visits to consultant-led mono specialty A&E service such as specialist emergency eye units and NHS walk-in centres. Emergency admissions were admissions that were ‘unpredictable and occur at short notice because of clinical need’, whereas elective admissions were defined as occurring when the ‘decision to admit can be separated in time from the actual admission’. We defined an outpatient attendance where a

For peer review only

child was recorded as having been seen by the intended care professional on the date of appointment on the HES outpatient appointments dataset (15). These outcomes are described in Supplementary File 1.

## Analysis

We calculated annual rates per 1000 child-years for each outcome for each financial year by summing the total number of events divided by the total child years of observation. To enable comparison across years, rates were age standardised to the 2016 mid-year English population aged<15 years (16). Similarly, annual rates were calculated for each developmental age-group from 2007 to 2017. However, due to poor HES A&E data quality before 2011, we only report ED visit rates from 2011/2012 (17). We calculated the change from baseline rate in 2007/8 to 2016/17, except for ED visits where we used a baseline reference year of 2011/12. We calculated the conversion proportion for emergency admission from 2011/2012 as the proportion of ED visits that led to emergency admissions.

## Patient and public involvement

Patients and the public were not directly involved in writing this paper.

# Results

Overall, 1,484,455 children from 408 practices contributed to this 10-year cohort study.
From 1 April 2007 to 31 March 2017, there were 7,604,024 GP consultations, 981,684 ED visits, 287,719 emergency admissions, 194,034 elective admissions, 2,253,533 outpatient visits among children aged less than 15 years in our population.

## GP consultations

The age standardised GP consultation rate dropped from 2005 to 1864 per 1000 child-years between 2007/8 and 2016/17 (Figure 1). In 2016/17, the telephone contact rates were 0.1 per child and home visits 1 per 1000 children The GP consultation rate in infants increased by 1%/ year from 6084 per 1000 child-years in 2007/2008. The GP consultation rate in pre-school children and those aged 5-9 years fell from 2707 to 2368 per 1000 child-years and 1416 to 1217 per 1000 child-years in the same study period respectively. For children aged above 10 years, the rate was stable throughout the decade (1100 per 1000 child-years) (Table 2).

For peer review only

Table 2 Annual rates of healthcare use per 1000 child-years and relative change from baseline 2007-2017[1,2]

| | Infants < 1 years | | | Preschool children 1-4 years old | | | School children 5-9 years old | | | Teenagers 10-14 years old | | |
|---|---|---|---|---|---|---|---|---|---|---|---|---|
| | Baseline | 2017 | % change[1] (95% CI) | Baseline | 2017 | % change[1] (95% CI) | Baseline | 2017 | % change[1] (95% CI) | Baseline | 2017 | % change[1] (95% CI) |
| **General practice consultations** | 6084 | 6722 | 10.5 (8.9, 12.0) | 2707 | 2368 | -12.5 (-13.2, -11.8) | 1416 | 1218 | -14.0 (-14.8, -13.2) | 1143 | 1074 | -6.0 (-7.0, -5.0) |
| **Outpatient visits** | 883 | 1529 | 73.1 (66.0, 80.2) | 518 | 706 | 36.3 (33.7, 38.9) | 477 | 649 | 36.2 (33.8, 38.6) | 451 | 648 | 43.9 (41.1, 46.7) |
| **Emergency department visits[2]** | 562 | 780 | 38.6 (32.8, 44.5) | 419 | 497 | 18.8 (17.0, 20.5) | 226 | 249 | 10.3 (8.2,12.3) | 280 | 297 | 6.2 (4.2, 8.1) |
| **Emergency admissions** | 312 | 410 | 31.6 (24.2, 39.1) | 109 | 123 | 12.8 (8.8, 16.8) | 38 | 39 | 2.8 (-2.9, 8.5) | 34 | 34 | -1.4 (-8.1, 5.2) |
| **Elective admissions** | 47 | 68 | 46.4 (14.1, 78.6) | 53 | 52 | -3.0 (-13.7, 7.6) | 48 | 47 | -2.4 (-10.3, 5.5) | 38 | 46 | 20.7 (-1.0, 42.3) |

1. % change calculated as (rate in 2017 – rate in baseline)/rate in baseline; 95% confidence interval (CI)
2. Baseline year used was 2007/2008 for all outcomes except emergency department visits which was 2011/2012 due to poor data quality poor prior to this time

For peer review

### Urgent and emergency care; ED visits and emergency hospital admissions

From 2011 to 2017, the age standardised ED visit rate increased by 1.6%/ year. There was a much larger increase of 3.9%/ year in annual ED visit rate for infants compared with older children. Since 2011, every year, 22% of ED visits led to an emergency admission. From 2007/2008 to 2016/2017, the age standardised emergency admission rate increased by 1.5%/ year from 75 per 1000 child-years and for infants by 3%/ year from 312 per 1000 child-years. However, the emergency admission rate in pre-school children and those aged above 5 years was stable throughout the decade (100-130 and <50 per 1000 child-years respectively).

### Planned care; outpatient visits and elective admissions

From 2007/8 to 2016/17, the annual age standardised outpatient visit rate increased from 508 to 724 per 1000 child-years (Figure 1). The outpatient visit rate in infants increased by 7.3%/ year over the study period. Rates in pre-school children and those aged above 5 years also increased steadily throughout the same period from 500 to 700 per 1000 child-years (Figure 2). Age standardised elective admission rates over the same study period were stable from 46 to 49 per 1000 child-years but increased by 4.6%/ year for infants and by 2%/ year for teenagers. Rates were stable for pre-schoolers and school children (~50 per 1000 child-years).

## Discussion

### Key findings

Infant contact rates with GPs have increased year on year over the past decade following major reforms in UK primary care but older children are using primary care less. GP consultations are the major point of contact with healthcare services across all age groups. Children's use of urgent care in hospitals, including visits to EDs and emergency admission rates, has increased particularly among infants. There have been sharp increases in planned care including outpatient activity and admissions for elective care in infants and teenage children.

### Comparison with previous studies

Health system failures to provide responsive care in the community to meet the needs of the children and families might well explain the rise in urgent care use. Parents prefer using their regular general GP when their child is unwell, but they choose to visit the ED if they perceive their child's condition as serious or cannot access primary care (18–20). It is estimated that 10% of infants attending the ED have no underlying medical problem (21), yet this does not mean that 90% need to seek emergency care if they require support to care for their child. The 2004 primary care healthcare reforms focused heavily on improving chronic disease management in adults with long term conditions and allowed primary care physicians to opt out of providing acute primary care services during evenings and weekends. This reduced access that may explain rising emergency department visits (3) . The expansion in the use of urgent care is consistent with trends in the UK and other high-income country settings where emergency admission rates in both children and adults have been rising for several years (7,22).

We have previously reported GP consultation rates for illness in children of a similar magnitude for a CPRD cohort born in 2000 and another study of healthcare use in primary care study that are remarkably consistent with our findings here for children (1,23). Infants, however, seem to be seeing their GP more frequently in 2017 compared with a decade ago. There is little research on children's outpatient service use and elective admissions but are consistent with overall rises in the total number of elective admissions, referrals to outpatient services and total outpatient attendances in English hospitals, which has increased markedly since 2007 (24–26).

For peer review only

Rising needs in the child population and demand from families are most likely contributors to the patterns of overall increasing healthcare use by children seen here (8). Preterm birth is increasing and is the single biggest cause of neonatal morbidity and mortality in the UK and other countries where survival rates are increasing (27). Thus, rising emergency admissions in infants could be explained by a growing morbidity burden in infants compared with other children. Recurrent admissions in children and young people with chronic conditions contribute substantially to total emergency admissions (28). A report projecting the State of Child Health in 2030 concluded that Britain's children who make up nearly 20% of the population have a rising health burden and poorer health compared with other wealthy countries. Even more concerning is a widening in the gap in health between wealthy and poor children (29). Hence growing morbidity burden particularly in deprived groups may be contributing to the rise in health care use.

The introduction of four-hour waiting target in ED in 2004 has been previously suggested as a contributing factor to increases in emergency admission rates (6,30). However, less than 5% of children's admission breach the 4-hour waiting time target so pressures within EDs are unlikely to explain the rising admission rate (31). Similarly, there is little evidence that ED staff have lowered their threshold for admitting children in our findings since the attendance admission ratio remained around 1 in 5 across the decade. Thus, our findings and previous studies point to mounting pressures in the community and illustrate the remarkable ability of hospitals in England to respond to the needs of families with young children.

## Strengths and Limitations

Our study is the first to report on trends in healthcare use by children for both primary and hospital care in the UK. This is among the largest studies to report healthcare use in children across the primary and secondary care interface in a health system with universal health coverage. The strengths of this study include its size, national coverage and representativeness of the UK population of both CPRD and HES data sources widely used for research. This reduces the possibility that our findings are due to chance. Few countries have health systems that enable the study of individual level data on children and links between primary care and hospital data.

However, there are several limitations to consider. First, in common with many observational studies using administrative records, data quality is variable. Our datasets did not include consultations in some urgent care settings including NHS walk in centres private providers. Use of HES A&E data was initiated in 2007/2008 but data quality and coverage did not improve until 2009. Hence we restricted our analysis of EDs to the period from 2011 onwards (17). HES Outpatient data were first available in 2003/2004 and have been validated for research from 2015/16.The rate of outpatient attendances in 2015/2016 has been ascribed to fluctuation in data quality and thus has not been reported in this paper (26). Second, we used a proxy for birthdate as CPRD only provides month of birth for children aged less than 15 years. This would overestimate the follow up period and underestimate the rate. Third, children living in deprived areas are more likely to deregister from CPRD practices than children residing in more affluent postcodes. Hence, our findings may not be generalisable to these groups (32). Finally, our findings may not be generalisable to certain patient groups missing from CPRD such as children using private health care services and homeless children (13).

## Implications and future research

The large sustained rises we report in primary care, urgent and planned care in hospital in infants have important implications for primary care. If rising urgent care use and falling contact rates with general practitioners are evidence of difficulties in children's access to GP appointments this is an important area for reform. It brings into question the continued selective financial incentive schemes for general practitioners to manage a rising multimorbidity burden in older patients. It is of concern that general practitioners are not universally trained in paediatrics and child health medicine as has been the recommendation of the European Academy of Paediatrics recently (33). Regardless, upwards trends in planned and urgent healthcare use by children have major resource and cost

For peer review only

implications for the NHS and we recommend the drivers and mediators of these changes warrant further research (3,34,35) . A further breakdown of data, which includes ethnic background, index of multiple deprivation, repeat attendance, presenting problems and case mix would be immensely helpful in understanding these trends. The number of health visitors and midwives who are providing support has decreased in England and Wales in recent years as a result of funding cuts (36). Accurate data on the effectiveness of this workforce in supporting parents is currently lacking but is recommended as an area for future research. Finally, there is scope to explore innovative approaches including parenting programmes and peer support for increasing parent's confidence and capacity in caring for their children.

## Conclusions

Over the past decade in England, general practice consultations have fallen for all children, except for infants. Children's use of hospital urgent and outpatient care has risen in all ages, especially infants. These changes may signify the need for better access to primary care and provision of specialist and community-based support for families with young children. Research to understand drivers and identify solutions to rising health service use is urgently needed.

For peer review only

## References


1. Hobbs FDR, Bankhead C, Mukhtar T, Stevens S, Perera-Salazar R, Holt T, et al. Clinical workload in UK primary care: a retrospective analysis of 100 million consultations in England, 2007–14. Lancet. 2016;387(10035):2323–30.
2. Shi Leiyu MJSB. Contribution of primary care to health systems and health. Milbank Q. 2005 Sep 1;83(3):457–502.
3. Cecil E, Bottle A, Cowling TE, Majeed A, Wolfe I, Saxena S. Primary Care Access, Emergency Department Visits, and Unplanned Short Hospitalizations in the UK. Pediatrics. 2016 Feb 1;137(2):e20151492.
4. Investing in General Practice The New General Medical Services Contract.
5. Roland M, Campbell S. Successes and Failures of Pay for Performance in the United Kingdom. Hamel MB, editor. N Engl J Med. 2014 May 15;370(20):1944–9.
6. Gill PJ, Goldacre MJ, Mant D, Heneghan C, Thomson A, Seagroatt V, et al. Increase in emergency admissions to hospital for children aged under 15 in England, 1999–2010: national database analysis. Arch Dis Child. 2013 May 1;98(5):328–34.
7. Cecil E, Bottle A, Sharland M, Saxena S. Impact of UK Primary Care Policy Reforms on Short-Stay Unplanned Hospital Admissions for Children With Primary Care–Sensitive Conditions. Ann Fam Med. 2015;13(3):214.
8. Keeble E, Kossarova L. Focus on: Emergency hospital care for children and young people. 2017.
9. Smith L, Narang Y, Ibarz Pavon AB, Edwardson K, Bowers S, Jones K, et al. To GP or not to GP: a natural experiment in children triaged to see a GP in a tertiary paediatric emergency department (ED). BMJ Qual Saf. 2017 Sep;bmjqs-2017-006605.
10. Gnani S, Morton S, Ramzan F, Davison M, Ladbrooke T, Majeed A, et al. Healthcare use among preschool children attending GP-led urgent care centres: a descriptive, observational study. BMJ Open. 2016 Jun;6(6):e010672.
11. The NHS plan : a plan for investment : a plan for reform : presented to Parliament by the Secretary of State for Health by command of Her Majesty : July 2000.
12. [ARCHIVED CONTENT] Improving coding, costing and commissioning - Audit Commission [Internet]. [cited 2019 Dec 13]. Available from: https://webarchive.nationalarchives.gov.uk/20111202180943/http://www.audit-commission.gov.uk/health/nationalstudies/pbr/pbr2011/Pages/pbr2011_copy.aspx#downloads
13. Herrett E, Gallagher AM, Bhaskaran K, Forbes H, Mathur R, Van Staa T, et al. Data Resource Profile: Clinical Practice Research Datalink (CPRD).
14. Mathur R, Bhaskaran K, Chaturvedi N, Leon DA, Van Staa T, Grundy E, et al. Completeness and usability of ethnicity data in UK-based primary care and hospital databases. J Public Heal (United Kingdom). 2014;36(4):684–92.
15. Hospital Episode Statistics Data Dictionary - NHS Digital [Internet]. [cited 2019 May 31]. Available from: https://digital.nhs.uk/data-and-information/data-tools-and-services/data-services/hospital-episode-statistics/hospital-episode-statistics-data-dictionary
16. Population estimates for the UK, England and Wales, Scotland and Northern Ireland - Office for National Statistics [Internet]. [cited 2019 Jun 7]. Available from: https://www.ons.gov.uk/peoplepopulationandcommunity/populationandmigration/populationestimates/bulletins/annualmidyearpopulationestimates/mid2016
17. HES 2011-12 A&E Data Quality Note Experimental data set. 2013.
18. Williams A, O'Rourke P, Keogh S. Making choices: why parents present to the emergency department for non-urgent care. Arch Dis Child. 2009 Oct 1;94(10):817–20.
19. Woolfenden S, Ritchie J, Hanson R, Nossar V. Parental use of a paediatric emergency department as an ambulatory care service. Aust N Z J Public Health. 2000 Apr 1;24(2):204–6.
20. Maguire S, Ranmal R, Komulainen S, Pearse S, Maconochie I, Lakhanpaul M, et al. Which urgent care services do febrile children use and why? Arch Dis Child. 2011 Sep 1;96(9):810–6.
21. Heys M, Kwong H-M, Reed J, Blair M. What do we really know about infants who attend Accident and Emergency departments? Perspect Public Health. 2014 Mar;134(2):93–100.
22. Health at a Glance 2015: OECD Indicators. Paris: OECD Publishing; 2015.
23. Cecil E, Bottle A, Ma R, Hargreaves DS, Wolfe I, Mainous AG, et al. Impact of preventive primary care on children's unplanned hospital admissions: a population-based birth cohort study of UK children 2000–2013. BMC Med. 2018 Dec 17;16(1):151.
24. Baker C. NHS Key Statistics: England, May 2019.
25. Dreyer K, Williamson RAP, Hargreaves DS, Rosen R, Deeny SR. Associations between

parental mental health and other family factors and healthcare utilisation among children and young people: a retrospective, cross-sectional study of linked healthcare data. BMJ Paediatr Open. 2018 Jul 30;2(1):e000266.
26. Hospital Outpatient Activity - NHS Digital [Internet]. [cited 2019 May 31]. Available from: https://digital.nhs.uk/data-and-information/publications/statistical/hospital-outpatient-activity
27. Blencowe H, Cousens S, Oestergaard MZ, Chou D, Moller A-B, Narwal R, et al. National, regional, and worldwide estimates of preterm birth rates in the year 2010 with time trends since 1990 for selected countries: a systematic analysis and implications. Lancet (London, England). 2012 Jun 9;379(9832):2162–72.
28. Wijlaars LP, Hardelid P, Woodman J, Allister J, Cheung R, Gilbert R. Contribution of recurrent admissions in children and young people to emergency hospital admissions: retrospective cohort analysis of hospital episode statistics. Arch Dis Child. 2015 Sep;100(9):845–9.
29. Viner R, Ward J, Cheung R, Wolfe I, Hargreaves D. Child health in 2030 in England: comparisons with other wealthy countries.
30. Saxena S, Bottle A, Gilbert R, Sharland M. Increasing Short-Stay Unplanned Hospital Admissions among Children in England; Time Trends Analysis '97–'06. van Baal PHM, editor. PLoS One. 2009 Oct;4(10):e7484.
31. Blunt I. Focus on : A & E attendances.Why are patients waiting longer. 2014.
32. Howe LD, Tilling K, Galobardes B, Lawlor DA. Loss to follow-up in cohort studies: bias in estimates of socioeconomic inequalities. Epidemiology. 2013 Jan;24(1):1–9.
33. Završnik J, Stiris T, Schrier L, Russell RR, del Torso S, Valiulis A, et al. Basic training requirements for health care professionals who care for children. Eur J Pediatr. 2018 Sep 25;177(9):1413–7.
34. Watson M, Blair M. Emergency departments and minor illness: Some behavioural insights. Archives of Disease in Childhood. 2018;103(4):309–10.
35. Shraim M, Blagojevic-Bucknall M, Mallen CD, Dunn KM. Repeated primary care consultations for non-specific physical symptoms in children in UK: a cohort study. BMC Fam Pract. 2014 Dec 5;15:195.
36. Buchan J, Charlesworth A, Gershlick B, Seccombe I. A critical moment: NHS staffing trends, retention and attrition. 2019.

For peer review only

# Additional Information

## Contributors:

SS and MB conceived and designed this study. JR prepared the data and carried out the statistical analysis overseen by EC and AB. JR, MB, EC, GG, AB, DH and SS took part in interpreting the data for this study, commented on and helped to revise drafts of this paper and have approved the final version. SS is the guarantor.

**Funding:** This article presents independent research supported by the National Institute for Health Research (NIHR) School for Public Health research (SS) the Collaborations for Leadership in Applied Health Research and Care (CLAHRC) programme for Northwest London (PD-SPH-2015-10055). The Dr Foster Unit at Imperial is affiliated with the National Institute of Health Research (NIHR) Imperial Patient Safety Translational Research Centre. The NIHR Imperial Patient Safety Translational Centre is a partnership between the Imperial College Healthcare NHS Trust and Imperial College London. The Dr Foster Unit at Imperial College are grateful for support from the NIHR Biomedical Research Centre funding scheme.

**Disclaimer:** The views expressed are those of the authors and not necessarily those of the NHS, the NIHR or the Department of Health and Social Care.

**Competing interests:** The authors declare that they have no competing interests.

**Patient consent:** Not required.

**Ethics approval:** We obtained ethical and scientific approval for the use of CPRD for our study from the Independent Scientific Advisory Committee Protocol number: 18_139.

**Provenance and peer review:** Not commissioned; externally peer reviewed.

**Data sharing Copyright information:** due to data sharing agreements, data are unavailable for sharing publicly.

**Copyright information:** This is an open access article distributed in accordance with the Creative Commons Attribution Non Commercial (CC BY-NC 4.0) license, which permits others to distribute, remix, adapt, build upon this work non-commercially, and license their derivative works on different terms, provided the original work is properly cited, appropriate credit is given, any changes made indicated, and the use is non-commercial. See: http://creativecommons.org/licenses/by-nc/4.0/.

**Figure legends**

Figure 1 Healthcare use in children aged less than 15 years

Figure 2 Healthcare use in children by developmental age groups

For peer review only

For peer review only

Figure 1 Healthcare Use in Children aged less than 15 years

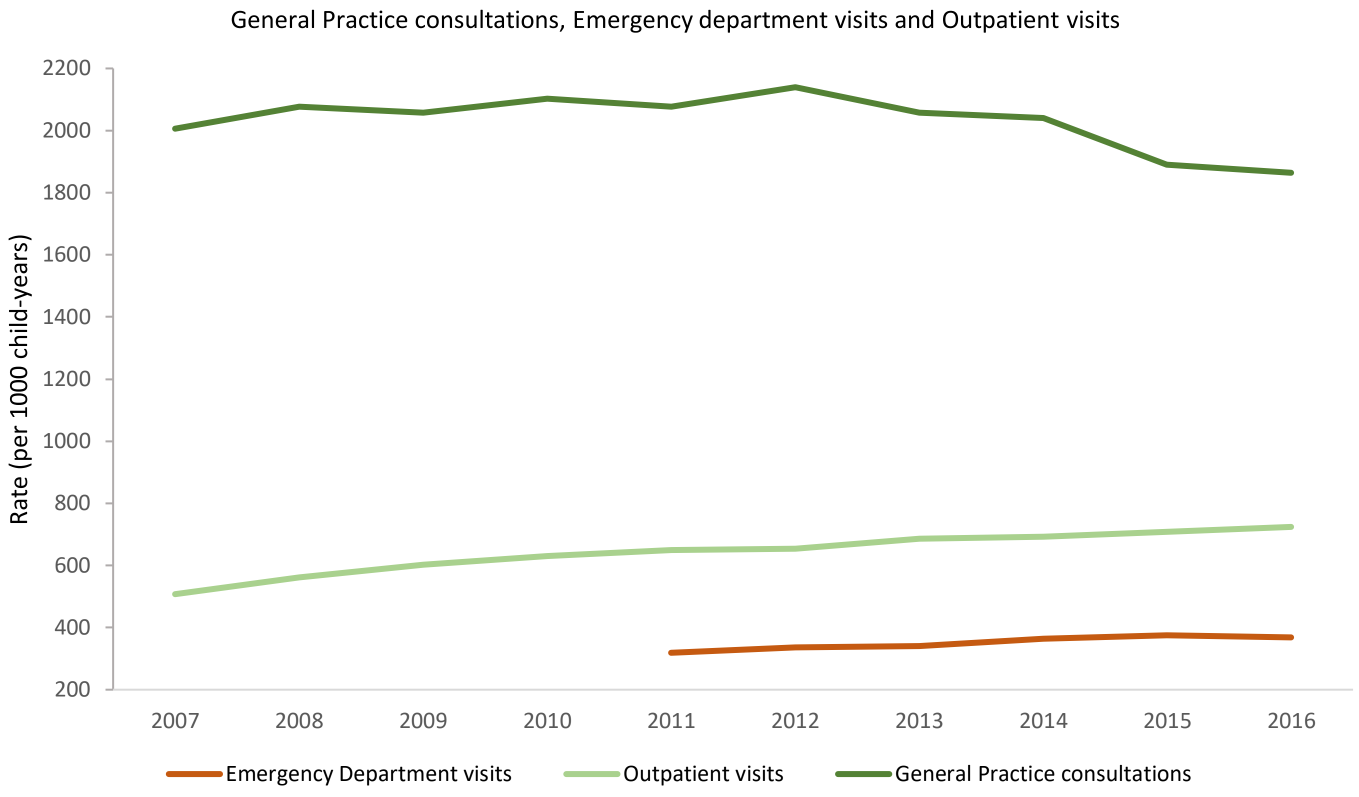


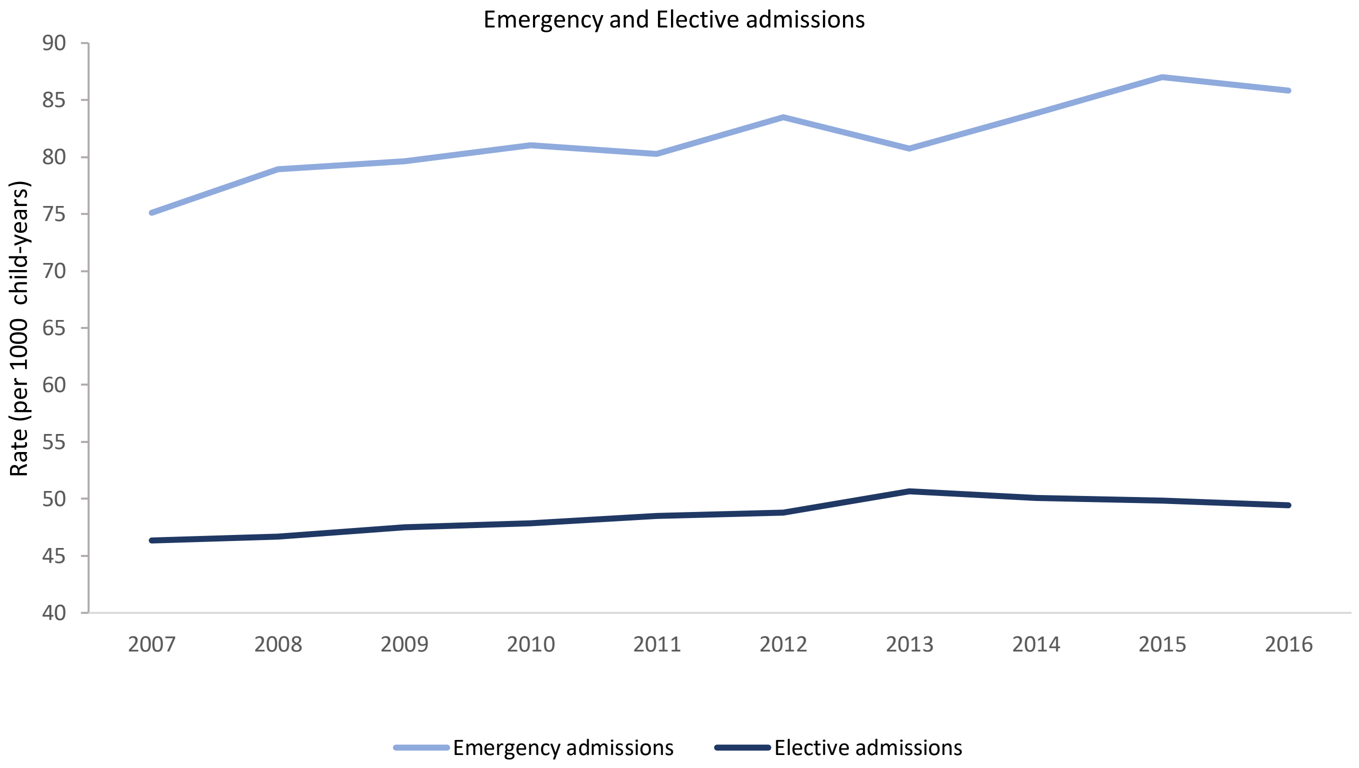


Rates were directly age-standardised to the 2016 mid-year English population

Figure 2 Healthcare use in children by developmental age groups

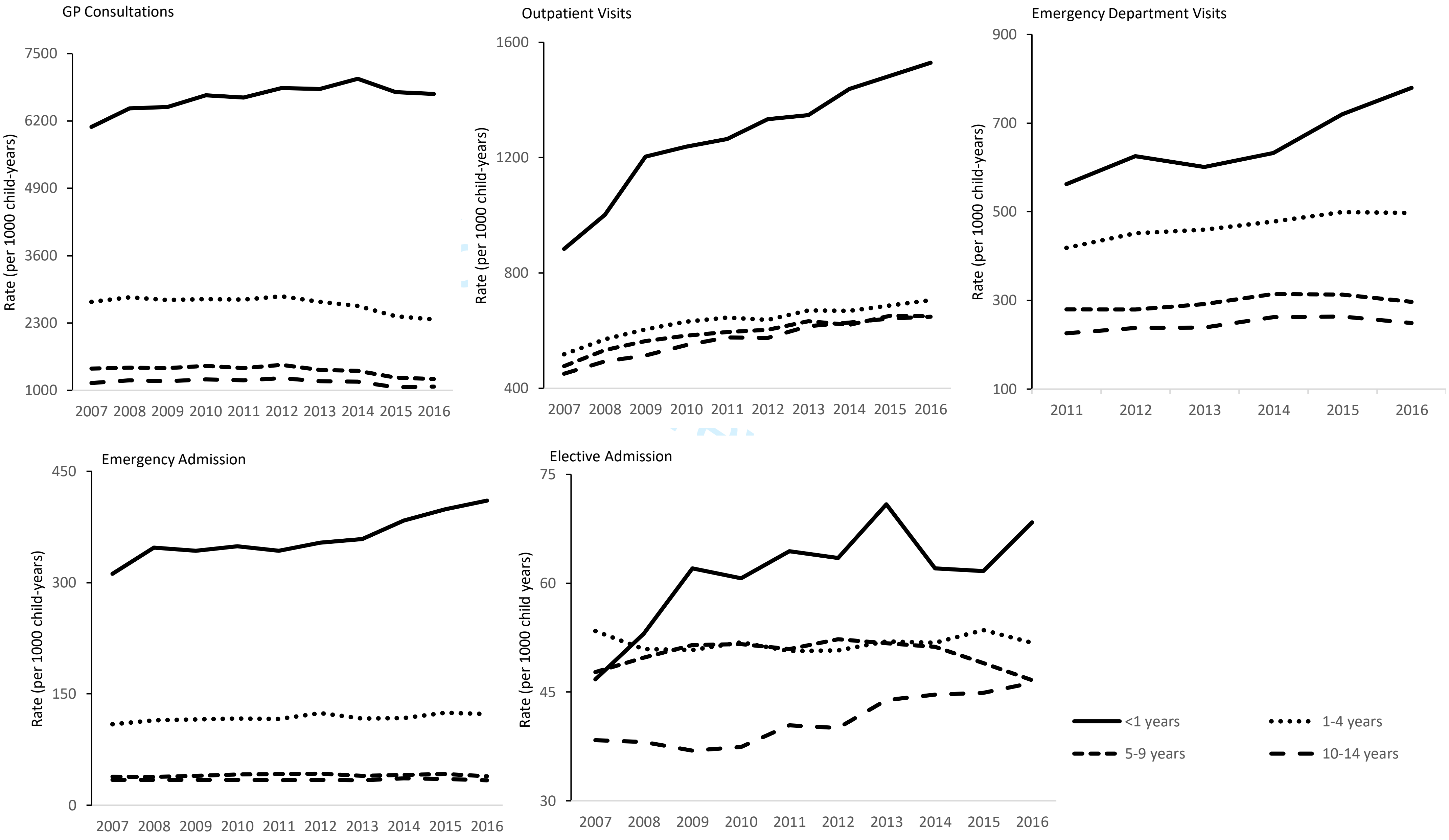

## Supplementary File 1

### Creating GP consultations

We defined a GP consultation as any face to face consultation for illness that took place on practice premises, excluding consultations for routine preventive care (immunisation and development checks).

Using CPRD clinical data we:

1) Defined a GP consultation as direct contact between a GP and a child using the consultation type look up table COT if the event date was recorded. Direct consultations were for Clinic, Follow-up/routine visit, Surgery consultation, Acute visit, Emergency Consultation and Co-op Surgery Consultation. Thus, selected Constype = (1, 3, 9, 11, 18, 36).

2) Differentiated between consultation with a GP and consultation with another practice member of staff by highlighting the staff who were GPs using the staff id and the Role of staff look up table ROL. Roles classified as GP were Senior Partner, Partner, Assistant, Associate, Locum, GP Registrar, Salaried Partner and GP Retainer. Thus, selected Role code = (1, 2, 3, 4, 7, 8, 47, 50).

3) Excluded records that were administrative using the Consultation type look up table SED. Thus, excluded type = administration (6).

4) We excluded GP consultations for preventive care (using the patient id and consultation ids isolated in preventive care records below). Thus, excluded consultations for immunisations, child development check and routine child health check.

### Supplementary Table 1: Read Codes identifying preventive care consultations

| **Description** | **V2 Read Codes** |
|---|---|
| **Preventive care** | |
| Child development checks | 64… |
| Routine child health check | ZV202 |
| Vaccinations | All vaccinations (from CPRD immunization files) |

### Creating Emergency Department (ED) visits

An ED visit was defined as an emergency department attendance at a consultant-led emergency department with 24-hour service, full resuscitation facilities and designated accommodation for the reception of accident & emergency (A&E) patients and into other types of A&E/minor injury department. We excluded visits to consultant-led mono specialty A&E service such as specialist emergency eye units and NHS walk-in centres.

Using HES Accidents & Emergency data we:

1) Defined an ED visit using the field 'AEDEPTTYPE' if 'ARRIVALDATE' was recorded. Thus, selected 'AEDEPTTYPE' = (1,3)

2) Defined source of referral using the field 'AEREFSOURCE'. Thus, selected self-referrals = 'AEREFSOURCE' = (1)

For peer review only

**Creating Emergency and Elective admissions**
Emergency admissions are unpredictable and occur at short notice because of clinical need whereas elective admissions are defined as occurring when the decision to admit can be separated in time from the actual admission.

Using HES Admitted Patient Care data we:
1) Defined an admission using the method of admission field 'ADMIMETH' if 'ADMIDATE' was recorded.

2) Differentiated between emergency admissions and elective admissions using the method of admission field 'ADMIMETH' below.

**Supplementary Table 2: ADMIMETH codes identifying emergency and elecive admissions**

| Outcome | ADMIMETH code used |
|---|---|
| Emergency admissions | 21, 22, 23, 24, 25, 28, 2A, 2B, 2C, 2D |
| Elective admissions | 11, 12, 13 |

**Creating outpatient visits**
We defined an outpatient attendance where a child was recorded as having been seen by the intended care professional on the date of appointment on the HES outpatient appointments dataset.

Using HES Outpatients data we:
1) Defined an attendance to Outpatients using the attended or did not attend field 'ATTENDED' if the field 'APPTDATE' for the appointment date was recorded. Thus, selected 'ATTENDED' = (5, 6).

For peer review only

# Annex 1: STROBE Statement—checklist of items that should be included in reports of observational studies

| | Item No | Recommendation | Pg. |
|---|---|---|---|
| **Title and abstract** | 1 | (*a*) Indicate the study's design with a commonly used term in the title or the abstract | 1 |
| | | (*b*) Provide in the abstract an informative and balanced summary of what was done and what was found | 2 |
| **Introduction** | | | |
| Background/rationale | 2 | Explain the scientific background and rationale for the investigation being reported | 4 |
| Objectives | 3 | State specific objectives, including any prespecified hypotheses | 4 |
| **Methods** | | | |
| Study design | 4 | Present key elements of study design early in the paper | 5 |
| Setting | 5 | Describe the setting, locations, and relevant dates, including periods of recruitment, exposure, follow-up, and data collection | 5 |
| Participants | 6 | (*a*) *Cohort study*—Give the eligibility criteria, and the sources and methods of selection of participants. Describe methods of follow-up<br>*Case-control study*—Give the eligibility criteria, and the sources and methods of case ascertainment and control selection. Give the rationale for the choice of cases and controls | 5 |

For peer review only

| | | | |
|---|---|---|---|
| | | *Cross-sectional study*—Give the eligibility criteria, and the sources and methods of selection of participants | |
| | | (*b*) *Cohort study*—For matched studies, give matching criteria and number of exposed and unexposed<br>*Case-control study*—For matched studies, give matching criteria and the number of controls per case | |
| Variables | 7 | Clearly define all outcomes, exposures, predictors, potential confounders, and effect modifiers. Give diagnostic criteria, if applicable | 5 |
| Data sources/ measurement | 8* | For each variable of interest, give sources of data and details of methods of assessment (measurement). Describe comparability of assessment methods if there is more than one group | *5* |
| Bias | 9 | Describe any efforts to address potential sources of bias | 5 |
| Study size | 10 | Explain how the study size was arrived at | N/A |
| Quantitative variables | 11 | Explain how quantitative variables were handled in the analyses. If applicable, describe which groupings were chosen and why | 6 |
| Statistical methods | 12 | (*a*) Describe all statistical methods, including those used to control for confounding | 6 |
| | | (*b*) Describe any methods used to examine subgroups and interactions | N/A |
| | | (*c*) Explain how missing data were addressed | 5 |
| | | (*d*) *Cohort study*—If applicable, explain how loss to follow-up was addressed | N/A |

For peer review only

| | | | |
|---|---|---|---|
| | | *Case-control study*—If applicable, explain how matching of cases and controls was addressed<br>*Cross-sectional study*—If applicable, describe analytical methods taking account of sampling strategy | |
| | | (*e*) Describe any sensitivity analyses | N/A |

**Results**

| | | | |
|---|---|---|---|
| Participants | 13* | (a) Report numbers of individuals at each stage of study—eg numbers potentially eligible, examined for eligibility, confirmed eligible, included in the study, completing follow-up, and analysed | 6 |
| | | (b) Give reasons for non-participation at each stage | N/A |
| | | (c) Consider use of a flow diagram | |
| Descriptive data | 14* | (a) Give characteristics of study participants (eg demographic, clinical, social) and information on exposures and potential confounders | N/A |
| | | (b) Indicate number of participants with missing data for each variable of interest | N/A |
| | | (c) *Cohort study*—Summarise follow-up time (eg, average and total amount) | N/A |
| Outcome data | 15* | *Cohort study*—Report numbers of outcome events or summary measures over time | *6* |
| | | *Case-control study*—Report numbers in each exposure category, or summary measures of exposure | |
| | | *Cross-sectional study*—Report numbers of outcome events or summary measures | |
| Main results | 16 | (*a*) Give unadjusted estimates and, if applicable, confounder-adjusted estimates and their precision (eg, 95% | 6-8 |

For peer review only

| | | | |
|---|---|---|---|
| | | confidence interval). Make clear which confounders were adjusted for and why they were included | |
| | | (*b*) Report category boundaries when continuous variables were categorized | |
| | | (*c*) If relevant, consider translating estimates of relative risk into absolute risk for a meaningful time period | |
| Other analyses | 17 | Report other analyses done—eg analyses of subgroups and interactions, and sensitivity analyses | N/A |
| **Discussion** | | | |
| Key results | 18 | Summarise key results with reference to study objectives | 8 |
| Limitations | 19 | Discuss limitations of the study, taking into account sources of potential bias or imprecision. Discuss both direction and magnitude of any potential bias | 9 |
| Interpretation | 20 | Give a cautious overall interpretation of results considering objectives, limitations, multiplicity of analyses, results from similar studies, and other relevant evidence | 8-9 |
| Generalisability | 21 | Discuss the generalisability (external validity) of the study results | 9 |
| **Other information** | | | |
| Funding | 22 | Give the source of funding and the role of the funders for the present study and, if applicable, for the original study on which the present article is based | 13 |

For peer review only
